# Supplementary material for: Driver or passenger? A new assessment of genes in the schizophrenia-associated 3q29 deletion locus for contribution to neurodevelopmental disorders
Source: J Neurodev Disord. 2026 Apr 21;18:39. doi: 10.1186/s11689-026-09696-y (PMC13326166; doi:10.1186/s11689-026-09696-y)
Supplement: Supplementary file 1 — Supplementary Material 1 [file 11689_2026_9696_MOESM1_ESM.docx]

Review article

**Driver or passenger? A new assessment of genes in the schizophrenia-associated 3q29 deletion locus for contribution to neurodevelopmental disorders**

Allyson R. Herriges^1^ and Ryan H. Purcell^1,2*^

^1^Fralin Biomedical Research Institute at Virginia Tech Carilion, Center for Neurobiology Research, Roanoke, VA, United States.

^2^School of Neuroscience, College of Science, Virginia Tech, Blacksburg, VA, United States.

*Correspondence: RHP (rpurcell@vtc.vt.edu)

**Abstract**

3q29 deletion (3q29Del) syndrome is caused by a 1.6Mb copy number variant (CNV) located near the telomeric end of the long arm of the third human chromosome. Hemizygosity of this set of 22 protein-coding genes significantly increases risk for schizophrenia and autism spectrum disorders among other neurodevelopmental conditions, but it is not known which genes in this CNV interval are responsible for these phenotypes. We have evaluated existing literature and public genomic resources for this set of genes, categorizing them based on known cellular functions and assessed their potential as phenotypic drivers. We provide a comprehensive, synthetic review of the essential known functions of 3q29 deleted genes, and how multiple 3q29-encoded proteins may functionally interact. Our analysis reveals that ubiquitination/SUMOylation stands out among processes potentially compromised due to compound haploinsufficiency of four 3q29Del genes (*UBXN7*, *FBXO45*, *RNF168*, *SENP5*). The available genomic evidence indicates that no single gene in the 3q29 locus is solely responsible for the neurodevelopmental phenotypes of 3q29Del syndrome. Overall, we propose that functional, expression, and gene constraint evidence supports six genes (*TFRC, UBXN7, FBXO45, PAK2, NCBP2, DLG1*) as the most likely phenotypic drivers in 3q29Del syndrome. Haploinsufficiency of these proteins would likely disrupt metabolic, synaptic, and signaling mechanisms in developing and mature neurons, which collectively may impair neural circuit differentiation and function.

**Introduction**

3q29 microdeletion (3q29Del) syndrome (OMIM # 609425) is a copy number variant (CNV) disorder that occurs in approximately 1:30,000-40,000 live births. This disorder is autosomal dominant, typically occurring *de novo*, and is caused by a hemizygous 1.6Mb microdeletion near the telomeric end of the long arm of human chromosome 3 [1, 2]. The deleted segment (~hg38 chr3:196,000,000 – 197,600,000) most commonly contains 22 protein-coding genes, but it is not known which of these genes, when hemizygously deleted, influence neural development or neuron and circuit function in the mature brain. Each of the 3q29Del genes may have a relatively consistent impact on brain development or physiology, or subsets of these genes may interact with one another as well as additional genes to yield a broader range of phenotypes. Indeed, 3q29Del-associated phenotypes vary widely from mild or subtle symptoms to major neurodevelopmental disability suggesting substantial interaction of 3q29Del genes [3, 4]. Nevertheless, these interactions as well as potential genetic or environmental modifying factors have not been identified. Understanding the developmental and neurobiological effects of 3q29Del could also have a broad impact as this CNV is the strongest known genetic risk factor for schizophrenia (SCZ) [5, 6], and may point to neurodevelopmental mechanisms of SCZ pathogenesis relevant to idiopathic cases.


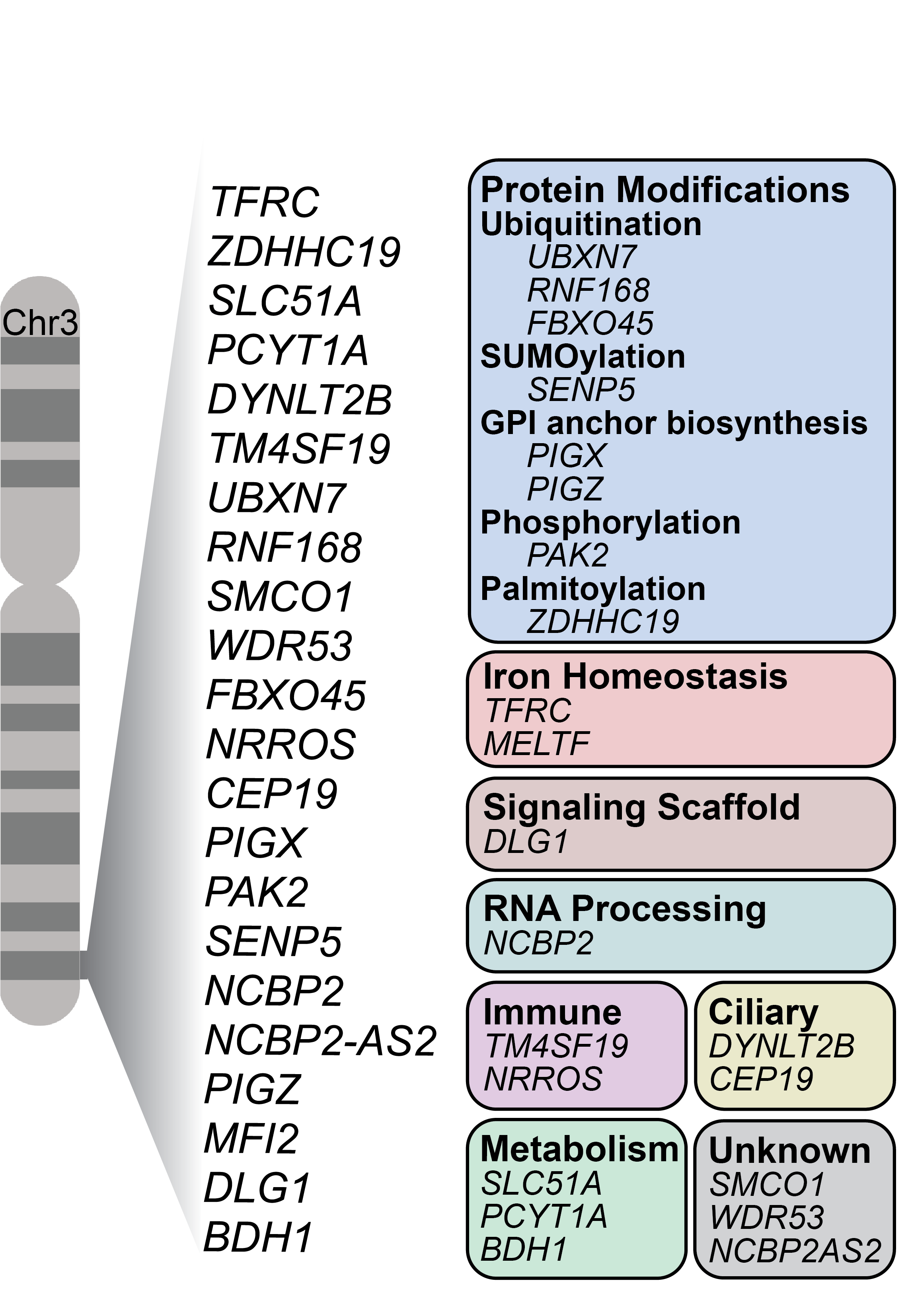


**Figure 1. Functional categorization of 3q29Del locus genes.** The genes in the 3q29Del locus are displayed in categories assigned based on known protein functions.

The natural history of 3q29Del syndrome remains an active area of investigation, but several detailed phenotyping studies have begun to provide insights. Infants with 3q29Del often experience failure to thrive [7], followed by delayed development and mild to moderate intellectual disability (ID) [8]. Clinical characteristics of 3q29Del often include neurodevelopmental and psychiatric conditions, with deletion individuals experiencing approximately 19x greater chance of autism spectrum disorder (ASD) diagnosis and a 40x increased likelihood of developing SCZ [9]. Other comorbid neuropsychiatric conditions include attention deficit/hyperactivity disorder (ADHD), generalized anxiety disorder (GAD), and graphomotor weakness. Additional common symptoms include sleep disturbance, congenital heart defects, gastroesophageal reflux disease, feeding difficulties, and ocular and dental anomalies [7, 10]. These phenotypes are often shared across neurodevelopmental anomalies [11]. The reciprocal 3q29 duplication has also been described and is associated with intellectual disability and risk for ASD [9]. While the 3q29 duplication is apparently less severe, these associations nonetheless further underscore the neurodevelopmental sensitivity to gene dosage at this locus.

Several genes within the 3q29Del interval (Fig. 1) have been individually associated with ASD (gene.sfari.org), such as *PAK2* [12]*, DLG1* [13]*,* and *TM4SF19* [14], but no studies have demonstrated comparable effect sizes to individually account for the entire risk conferred by 3q29Del. Oligogenic CNVs can be more complex than single-gene variants, but may be a more tractable way to understand the polygenic nature of complex neuropsychiatric conditions [15]. Previous studies of other variants have demonstrated that CNVs can elicit phenotypes in several ways [16]. Haploinsufficiency of a single driver gene within the deletion locus may account for all or most phenotypes as appears to be the case for *SHANK3* at 22q13 (OMIM # 606232) [17, 18]. Alternatively, one gene may be associated with a certain phenotype and another gene may be responsible for a separate phenotype—sometimes referred to as “contiguous gene effects”—as in the Williams-Beuren syndrome (OMIM # 194050) locus (7q11.23). In 7q11.23 deletion, haploinsufficiency of the gene *Gtf2ird1* was found to produce the auditory hyperacuity phenotype in mice [19] whereas loss of *ELN* causes the arterial disease supravalvular aortic stenosis [20]. Another possible mechanism is that compound haploinsufficiency of multiple genes within a CNV locus (to varying degrees) produces a phenotype. The craniofacial and neuroanatomical abnormalities that result from copy number variation at 16p11.2 and 22q11.2 seem to be produced by multiple genes in this manner [21, 22].

To date, genomic studies do not support the hypothesis that any single gene within the 3q29Del locus is responsible for the entire phenotype and analysis of single gene loss-of-function or heterozygous mutations in multiple animal models have sought to determine which genes may be the primary phenotypic drivers. For example, mice heterozygous for the 3q29Del gene *Dlg1*, a candidate gene that encodes a multi-function synaptic signaling scaffold protein, did not recapitulate phenotypes of the full syntenic 3q29Del mouse which include spatial memory and social interaction deficits [23]. A combinatorial knockdown of *Drosophila* homologs demonstrated that diminished expression of the 3q29Del gene *NCBP2*, which encodes an mRNA cap-binding protein, exacerbates phenotypes associated with losses of other 3q29 genes [24]. A bioinformatic analysis of the human cortical transcriptome indicated that the 3q29Del gene *UBXN7* may play a similar role as a cortical “hub gene” [25]. None of these three candidate genes can explain the entirety of ASD or SCZ risk conferred by 3q29Del.

Beyond top candidates, many of the genes in this locus remain poorly understood [25]. Revisiting available literature in combination with bioinformatic analyses provides insight into likely phenotypic disruptions due to 3q29Del. Clues as to which 3q29 genes may be essential for neurodevelopment could be uncovered through analysis of genomic data from healthy individuals. Thus, we have leveraged the publicly available Genome Aggregation Database (gnomAD) and the Allen Institute’s BrainSpan developmental atlas to analyze mutational constraint metrics and brain-specific mRNA expression for each protein-coding gene in the 3q29Del interval. The Loss-of-function Observed/Expected Upper bound Fraction (LOEUF) is a continuous variable estimate that reflects an approximation of the constraint faced by each gene. Smaller values indicate that less missense and nonsense variation is observed in genes than expected based on synonymous variation rates [26, 27], consistent with negative selection for loss or gain of function variants. Low LOEUF scores can be indicative of broad essentiality but does not specifically indicate relevance to neurodevelopment. When paired with the neurodevelopmental specificity of the BrainSpan database, a moderate-to-low LOEUF score with moderate-to-high levels of RNA expression suggests that the gene is both intolerant to loss-of-function variation and involved in neurodevelopmental processes, thus increasing the likelihood of being a phenotypic driver in 3q29Del. (Fig. 2, Table 1). This multifactorial analysis based on mutational constraint, neurodevelopmental expression levels, functional annotations, and computational estimates of haploinsufficiency [28] has led us to group these 22 genes into 4 Tiers. Tier 1 genes (6) are likely phenotypic drivers based on multiple lines of evidence. Next are those genes that are likely (Tier 2, 7 genes) or possibly (Tier 3, 3 genes) involved as modifiers of certain phenotypes. We have found no compelling evidence to support the involvement of the remaining 6 genes.

Our bioinformatic analysis of 3q29Del genes as well as re-assessment of the literature on function of individual genes based upon mutant analyses in multiple models provides an up-to-date description of 3q29Del gene functional categories based on their inferred primary or obligate functions as was previously done for the neuropsychiatric disorder-associated CNV 22q11.2 [29]. These functional categories are aligned to possible cellular mechanisms that impact neural development and may therefore indicate neurodevelopmental pathways that are vulnerable to 3q29Del, which can be prioritized for experimental investigation.

**Protein Modifications**

*Ubiquitination and SUMOylation*

Ubiquitination stands out as a broad cellular process that may be compromised by haploinsufficiency of multiple 3q29 genes. Ubiquitin and ubiquitin-like proteins (such as SUMO) are eukaryotic post-translational modifications that are essential for many cellular processes including regulation of protein homeostasis [30]. Interestingly, four genes within the 3q29Del interval are involved in this process and, while it is not yet clear if any genetic interactions exist between these genes, the ubiquitin-like system may be a point of vulnerability in 3q29Del. Numerous studies have demonstrated critical roles of other ubiquitination proteins in neuronal development [31]. For example, the E3 ubiquitin ligase encoded by *UBE3A* (15q11.2) illustrates a key connection between the ubiquitination system and neurodevelopmental disorders. Alterations in the dosage of this gene results in Dup15q syndrome (OMIM #608636) [32, 33], Angelman Syndrome (OMIM #105830), or Prader-Willi Syndrome (OMIM #176270), which are all associated with neurodevelopmental disorders [34]. Ube3a has been found to be required for experience-dependent plasticity and visual cortex circuit development [35]. Furthermore, genes encoding members of the Cullin family of E3 ligases have also been recently associated with neuropsychiatric risk and are discussed further below [6, 36, 37].

**UBXN7 (Tier 1)**

*UBXN7 (aka UBXD7)* encodes the UBA-UBX domain protein family member UBXN7 [38], which is widely expressed across numerous tissues including the brain. As a UBA-UBX protein, UBXN7 can function as an adaptor for interacting with ubiquitinated proteins through its UBA domain, and to bind via its UBX domain to the p97 ATPase, which is involved in diverse cellular functions such as protein homeostasis, membrane remodeling, and chromatin regulation [39].


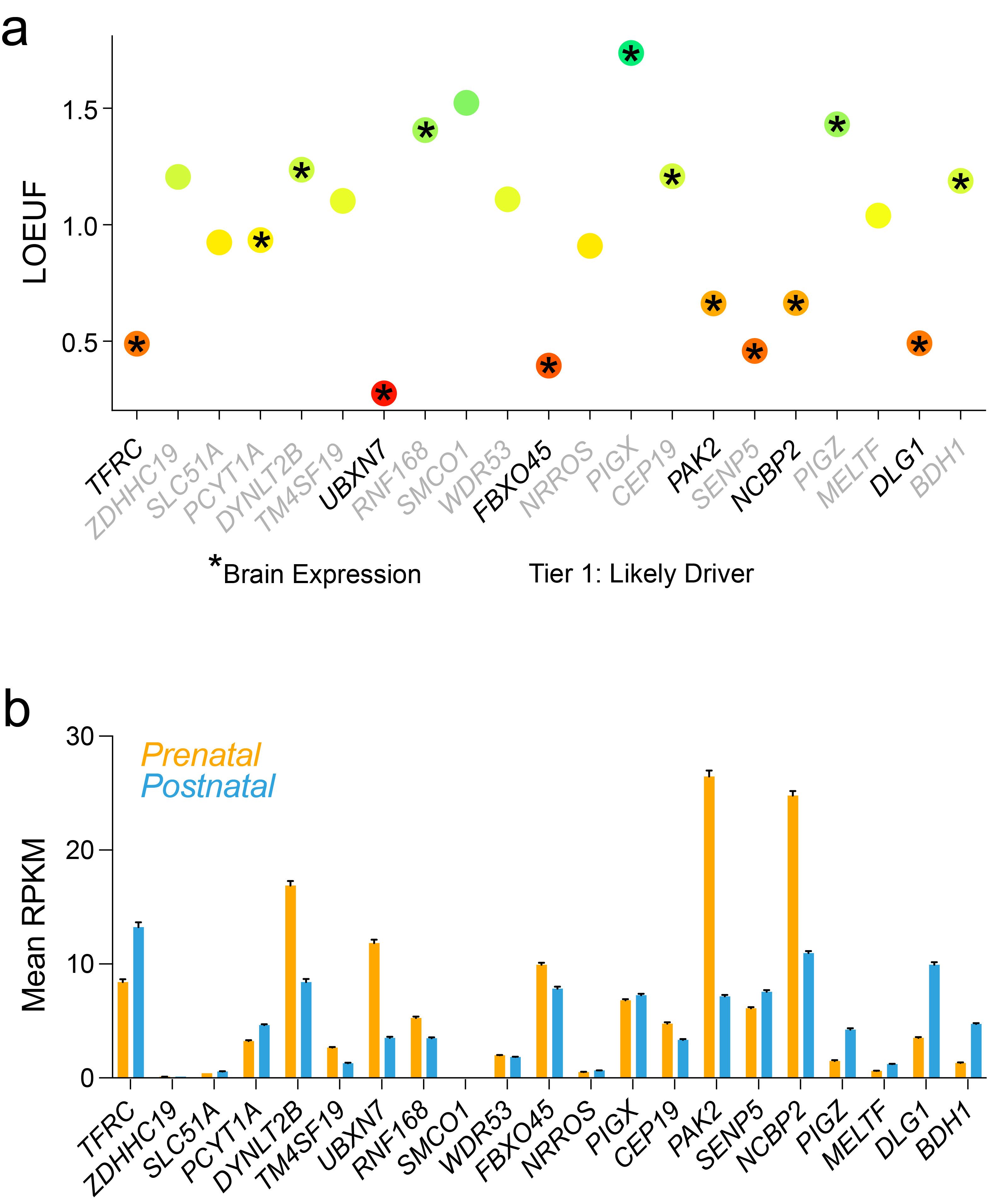


**Figure 2. Mutational constraint and brain expression of 3q29Del genes.** (**a**) The Loss-of-function Observed/Expected Upper bound Fraction (LOEUF) is plotted for each 3q29Del locus gene arranged by chromosomal position (centromere to telomere) from the Genome Aggregation Database (gnomAD) version 4.1. Asterisks indicate brain expression ascertained from BrainSpan [53] (**b**). Data in **b** are mean RPKM + SEM across brain region samples and individuals from BrainSpan.

UBXN7 has been found to play a key role in the regulation of cellular hypoxia and antioxidant responses. The master regulator of the hypoxia response, HIF1α, is constitutively degraded in normoxic conditions by the CUL2^VHL^ E3 ubiquitin ligase complex [40]. UBXN7 was found to recruit ubiquitinated HIF1α to p97 to facilitate its degradation [41]. A lack of UBXN7 in 3q29Del may therefore be expected to compromise the cellular hypoxia response. Additional studies have found that UBXN7 is a promiscuous interactor with CUL1, CUL2, and CUL3-based E3 ubiquitin ligases [42]. These interactions are notable as rare variants in *CUL1* were found to confer high risk for schizophrenia [6] and *CUL3* has been strongly associated with risk for ASD [36, 37]. Moreover, CUL3 is part of the complex that regulates cellular levels of the antioxidant response protein NRF2 [43] whereas CUL2 regulates HIF1α [44]. UBXN7 binds to both complexes and the cellular protein level of UBXN7 has been found to dictate the balance of NRF2 [45] and HIF1α in immortalized cell lines [46].

Levels of UBXN7 are regulated by an E3 ubiquitin ligase called MUL1 [46] that is localized to the outer mitochondrial membrane and can signal mitochondrial status [47]. When MUL1 is inactive, levels of UBXN7 increase, which promotes HIF1α accumulation and a shift toward glycolysis to meet cellular energy demands [48]. However, when UBXN7 levels are diminished, as is the case in 3q29Del, NRF2 levels may increase, HIF1α levels may decrease, and cells shift toward mitochondrial oxidative phosphorylation (OxPhos) to meet energy needs [46]. These NRF2-HIF1α experiments were performed in engineered human embryonic kidney cells, and it is not known how 3q29Del would impact these pathways, particularly in the developing central nervous system. These studies are intriguing in light of the recent finding that 3q29Del human neural progenitor cells do not switch from glycolysis to OxPhos like intact cells from neurotypical controls [49]. UBXN7 may be predicted to be involved in the critical metabolic reprogramming phase of neurogenesis and neuronal differentiation.

A genetic screen aimed at identifying proteins with a role in maintaining stem cell identity through mitosis revealed that UBXN7 is involved in recruiting the p97 ATPase to ubiquitinated histones for rapid histone degradation and initiation of cell-type specific transcriptional programs [50], which may compromise the stability of the neural stem/progenitor cell state. Precocious neuronal differentiation from neural stem/progenitor cells has been described in several models of neurodevelopmental disorders including Fragile X Syndrome [51] and 16p11.2 deletion [52]. It is not yet known if haploinsufficiency of UBXN7 in 3q29Del would similarly impact neural differentiation trajectories.

Lastly, *UBXN7* has the lowest LOEUF score of any 3q29Del gene (Fig. 2), and is expressed in human brain at particularly high levels prenatally [53]. Thus, *UBXN7* is a strong candidate as a phenotypic driver gene and may be involved in multiple aspects of 3q29Del associated neurodevelopmental phenotypes.

**RNF168 (Tier 2)**

*RNF168* encodes an E3 ubiquitin ligase that plays a critical role in DNA double-strand breaks (DSBs) [54–56]. In the case of a DSB, RNF168 cooperates with RNF8 to ubiquitinate histone proteins and, like UBXN7 above, recruits the ATPase p97 to the site, though for a different purpose [57]. Homozygous loss-of-function mutation of RNF168 results in RIDDLE syndrome, a human immunodeficiency disorder caused by dysfunctional DNA repair [58], though it is not clear if hemizygosity of *RNF168* (as in 3q29Del) is associated with known phenotypes. *RNF168* has also been identified as a candidate gene for both Parkinson’s disease [59] and Crohn’s disease [60]. RNF168 has not been found to be significantly reduced at the protein level in 3q29Del mice due to apparent post-transcriptional compensation [23] and LOEUF analysis and relatively low expression in developing and adult brain indicates it is unlikely to be a phenotypic driver in the hemizygous state. However, due to its involvement in ubiquitination pathways, hemizygotic effects of *RNF168* may be amplified by loss of other 3q29 genes.

**FBXO45 (Tier 1)**

*FBXO45* encodes a member of the F-box protein family, that typically functions as an E3 ubiquitin ligase via the formation of SCF (Skp1, Cullin, F-box) complexes [61]. However, FBXO45 was found to not form SCF complexes and instead binds to the RING finger-type ubiquitin ligase PAM (aka MYCBP2) [62]. One ubiquitination target of the FBXO45 complex was reported to be the conserved pro-apoptotic transcription factor p73 [63]. Biochemical subcellular fractionation, immunocytochemistry, and immunoelectron microscopy experiments demonstrated that Fbxo45 localizes to pre- and post-synaptic compartments of neurons [64]. In mice, Fbxo45 protein was found almost exclusively in the nervous system. While *Fbxo45* heterozygous mice showed no phenotypic differences from their wild type counterparts, full knockout of *Fbxo45* resulted in perinatal lethality due to respiratory distress [62]. Fbxo45-null mice also showed significant abnormalities in synapse formation, diaphragm innervation, axon development, and neuronal migration indicating that the protein could have a key role in neurodevelopment [62]. Knockdown of Fbxo45 in hippocampal neurons increased excitatory postsynaptic currents, possibly due to a disruption of Fbxo45-mediated ubiquitination of the synaptic vesicle-associated protein Munc13-1 [64]. In addition to its role in ubiquitination, Fbxo45 was surprisingly found to be secreted and to bind to N-cadherin [65].

The synaptic localization of FBXO45 and its link to the Tuberous Sclerosis Complex proteins via PAM/MYCBP2 [66] prompted sequencing of *FBXO45* in an ASD trio study, but no variants were identified that were over-represented in ASD probands [67]. A sequencing study in individuals with SCZ found one rare variant in *FBXO45* [68], though this variant has not been identified in larger, subsequent studies [6]. A genome-wide association study reported that *FBXO45* may also have a role in emotional regulation [69].

Based on its synaptic localization and essential role in mammalian CNS development *FBXO45* is an intriguing candidate driver gene. Gene constraint analysis from gnomAD indicates that it is in the top decile of the most constrained human genes and ranks second in the 3q29 locus (to *UBXN7*), and thus likely to be haploinsufficient. Further studies are needed to understand how loss of one copy of this gene may disrupt neurodevelopment in 3q29Del syndrome.

While each of these three proteins appear to handle largely distinct roles in ubiquitination, it is likely that we do not understand the full range of their functions, which may overlap in critical ways in specific cell types or states at certain phases of development.

**SENP5 (Tier 2)**

*SENP5* encodes a protease for the SUMO protein (small ubiquitin-like modifier) that is involved in mitosis [70, 71]. SUMO proteins are covalently attached as a post-translational modification, which can be reversed by SUMO-specific proteases such as SENP5 [72]. SUMOylation impacts numerous cellular processes like DNA repair, protein stabilization, apoptosis, and signal transduction [73]. *SENP5* knockdown by shRNA was found to alter mitochondrial morphology and increase reactive oxygen species production [67]. Further investigation revealed that SENP5 shuttles to mitochondria during cell division and is involved in mitochondrial fragmentation [74]. SENP5 is not among canonical mitochondrial proteins [75], and this cell cycle-dependent translocation behavior may explain why. Mitochondrial fragmentation is also an essential phase of apoptosis, which involves SUMOylation of the mitochondrial fission protein DRP1 [76]. Increased SUMOylation of DRP1, which could result from a lack of SENP5, would increase the likelihood of initiating apoptotic signaling cascades [77]. Interestingly, the E3 ubiquitin ligase MUL1 (aka MAPL), which was found to ubiquitinate and regulate levels of UBXN7 (see above), also functions as an E3 SUMO ligase that SUMOylates DRP1 [77]. The effect of haploinsufficiency of both *UBXN7* and *SENP5* on mitochondrial dynamics and function is not clear.

An examination of Senp proteins Senp3 and Senp5 in mouse brain found that Senp3 expression is restricted to cell nuclei whereas Senp5 was found in the neuropil and to co-localize with presynaptic, postsynaptic, and mitochondrial markers [78]. Further studies found that the balance between two Senp5 splice variants, Senp5L and Senp5S, regulates the balance of mitochondrial fission/fragmentation as the smaller “S” isoform lacks the protease catalytic domain and competes with full-length Senp5 for substrate binding [79]. Additionally, *in vivo* knockdown of *Senp5* was found to disrupt the radial migration of neurons into the cortical plate. These findings suggest that *SENP5* could be involved in 3q29Del phenotypes and that compound hemizygosity of *SENP5* and *UBXN7* could be more deleterious than loss of one copy of either gene alone.

*Phosphorylation and Signaling*

**PAK2 (Tier 1)**

PAK2 is a ubiquitously expressed serine/threonine protein kinase that is crucial to numerous signaling pathways such as apoptosis [80], cell division, cytoskeleton regulation, and cell motility [81]. Interestingly, BrainSpan data shows very high prenatal expression of *PAK2* (Fig. 2b), especially at very early phases of neurodevelopment (Supp. Fig. 1) that diminishes postnatally.

PAK2 is typically activated by small GTPases such as Rac1 and Cdc42 [82], but can also be activated by the TSC and mTOR-related small GTPase Rheb [83]. Heterozygous *Pak2* knockout in mice resulted in autism-like behaviors, defective long-term potentiation (LTP), and reduced spine density [12], and it is now considered a strong ASD candidate gene. Notably, heterozygous *Pak2^+/-^* deletion did not recapitulate all behavioral phenotypes of 3q29Del mice [23, 84]: spatial memory and startle responses appeared to remain intact. However, diminished Pak2 expression in cortex and hippocampus of *Pak2^+/-^* mice was associated with reduced phosphorylation of Limk1 and Cofilin-1, which are key regulators of dendritic spine cytoskeletal dynamics and possibly underlying the reported reduction in hippocampal dendritic spines and theta burst-induced LTP [12]. LIMK1, which is directly activated by PAK2 [85], is encoded by *LIMK1* in the Williams-Beuren Syndrome locus 7q11.23 (deletion), illustrating a potential molecular link between these CNV syndromes that are both associated with social differences.

PAK2 also has an emerging connection to cellular and organismal energy homeostasis. This metabolic role for PAK2 could be related to direct phosphorylation by the glycolytic enzyme pyruvate kinase M2 [86] and the cellular energy balance sensor AMPK [87, 88]. Fasting increases AMPK-mediated PAK2 phosphorylation in the mouse arcuate nucleus of the hypothalamus, a crucial food intake signaling hub [88]. At the cellular level, genetic or pharmacologic disruption of PAK2 can shift cells toward more oxidative phosphorylation-based metabolism [89] and overexpression of *PAK2* can promote aerobic glycolysis and cell proliferation [90]. The full 3q29Del was found to impair metabolic flexibility in developing neural progenitor cells and complete knockout of *PAK2* in HEK cells partially recapitulated that phenotype [49].

While complete knockout of *Pak2* in mice results in embryonic lethality due to neural tube defects [81, 91], Schwann cell-specific knockout was found to severely disrupt myelination, decrease nerve conduction velocity, and produce peripheral nerve dysfunction [92]. A biallelic *PAK2* missense mutation was linked to Knobloch Syndrome type 2, a rare disorder that presents with severe vision problems and craniofacial malformation [93]. PAK2 has also been linked to developmental language disorder [94], cardiac, inflammatory, and immunological diseases [95]*,* and is an autosomal homolog of *PAK3,* a gene linked to neurodevelopmental delays and behavioral abnormalities [96]. The LOEUF score for *PAK2* is moderate at 0.662 but findings from the heterozygous mice indicate that heterozygous loss of *Pak2* alone disrupts neurodevelopment and therefore is likely involved in at least some phenotypic manifestations of 3q29Del Syndrome.

**DLG1 (Tier 1)**

*DLG1* encodes a ubiquitously expressed, large, multi-functional protein necessary for multiple domains of development [97]. DLG1 (aka SAP97) is widely expressed in various cellular compartments and throughout many tissues, but is notably found at synapses and is involved in the stabilization and maturation of newly-formed dendritic spines [98]. As a member of the Membrane-Associated Guanylate Kinase (MAGUK) family, DLG1 can act as a signaling hub facilitating the interactions of multiple proteins including cell surface receptors and effector molecules. DLG1 has three PDZ (PSD95/DLG/ZO-1) domains and has been reported to bind to G protein-coupled receptors with PDZ binding intracellular C-termini including the β1-adrenergic receptor [99], ADGRB1 (BAI1) [100, 101], ADGRA3 (GRP125) [102], ADGRE5 (CD97) [103], the serotonin 5-HT2A receptor [104], the corticotropin-releasing factor 1 receptor [105], and Frizzled-4 [106]. These interactions open the possibility that reductions in DLG1 protein resulting from 3q29Del [23] could impact numerous signaling cascades relevant to neurodevelopment and adult circuit function.

The *DLG1* gene produces multiple isoforms, which have differential effects on synaptic plasticity [107]. Indeed, DLG1 protein interacts with subunits of key glutamate receptors involved in synaptic plasticity. It binds to the NR2A subunit of NMDA receptors via one of its three PDZ domains. NMDA receptor activation promotes DLG1 phosphorylation by CaMKII, which disrupts the DLG1-NR2A interaction [108]. CaMKII phosphorylation also disrupts the interaction between DLG1 and the astrocytic glutamate transporter EAAT2 [109], possibly indicating a generalized mechanism of interactions.

DLG1 binds to the AMPA receptor subunit GluR1 and the complex travels together from the endoplasmic reticulum to postsynaptic compartments [110, 111]. DLG1 L27 domain-mediated interaction with the X-linked neurodevelopmental disorder associated protein CASK (calcium/calmodulin-dependent serine protein kinase) influences DLG1 conformation and interaction with AMPA or NMDA receptor subunits [112, 113]. In addition to glutamate receptor subunits, which form Na^+^ and Ca^2+^ channels, DLG1 interacts with the inward rectifier potassium channel Kir2.2 [114] and the voltage-gated potassium channel Kv4.2 at the post-synaptic density [115]. DLG1 is found in both pre- and post-synaptic compartments and, interestingly, postsynaptic expression of Dlg1 was found to affect presynaptic function [116]. DLG1 has also been found to have key non-synaptic roles in neural function. For example, DLG1 interacts with PTEN to inhibit AKT and serve as a brake on peripheral axon myelination [117].

For a ubiquitously expressed, multi-functional protein like DLG1, the cellular context is critically important for understanding specific functional roles. For example, a recent study found that knockdown of *Dlg1* expression had no measurable effect on hippocampal area CA1 neurotransmission but dramatically increased AMPA receptor-mediated activity in the dentate gyrus [118]. Thus, whole-body heterozygosity of *DLG1* as in 3q29Del may only elicit phenotypes in certain neural cell types and circuits.

Given the large interactome of DLG1 and its formation of signaling complexes, it has been hypothesized that DLG1 may interact directly or indirectly with other 3q29Del locus proteins. Indeed, there is suggestive evidence of a genetic interaction between *DLG1* and *PAK2* in a *Drosophila* model. Heterozygous deletion of either homolog *dlg* or *pak* did not elicit an observable phenotype but *dlg/pak* transheterozygotes were found to have disrupted sleep patterns and a loss of neuromuscular junction synapses [119].

The direct connection of *DLG1* to schizophrenia risk, however, remains unclear. Among 3q29Del locus genes, *DLG1* has received by far the most attention as a potential individual driver of neuropsychiatric disease risk, likely due to the extensive literature describing the role of DLG1 at synapses and the link between synaptic genes and schizophrenia risk [120, 121]. Notably, a *Dlg1* heterozygous mouse was found to not recapitulate the behavioral phenotypes of the 3q29Del mouse model [23]. Rare, *de novo* mutations in *DLG1* have been found in cohorts of individuals with schizophrenia [122] though larger, genome-wide analyses have not implicated rare *DLG1* variants. Targeted analysis of single-nucleotide polymorphisms (SNPs) in *DLG1* have identified several variants enriched in schizophrenia cohorts, but it is unclear if these loci meet genome-wide significance thresholds [123–126]. The most recent schizophrenia GWAS found a SNP linked to *DLG1* to be associated with modest (~3% increase) but significant risk for SCZ, though the impact of this SNP on gene expression or protein function has not been reported [121]. Genomic (LOEUF) evidence indicates that *DLG1* may be mutationally constrained, though the complexity of its many isoforms and extensive protein-protein interactions could complicate this analysis. Interestingly, BrainSpan data indicates that *DLG1* expression is rather modest prenatally and increases postnatally (Fig. 2). Together, the evidence suggests that *DLG1* is likely involved as a phenotypic driver gene though haploinsufficiency of *DLG1* alone is not responsible for the entirety of 3q29Del syndrome.

*GPI Anchor Biosynthesis*

**PIGX** and **PIGZ (Tier 2)**

Two Phosphatidyl Inositol Glycan genes, *PIGX* and *PIGZ* are found in the 3q29Del interval, both of which encode proteins that are involved in mannose attachment to glycosylphosphatidylinositol (GPI) anchors and are expressed during brain development (Fig. 2). *PIGX* encodes a subunit of the GPI mannosyltransferase complex. GPI is a glycolipid that anchors numerous proteins to the cell surface [127]. Within the GPI complex, the function of PIGX is to stabilize the PIGM enzyme [128].

*PIGZ* is responsible for attaching a fourth mannose to the GPI complex. mRNA analysis has revealed that *PIGZ* is expressed across most human tissues, with the highest levels found in the brain and colon [129]. Mouse knockout strains of *PIGZ* resulted in increased levels of amyloid beta peptide, a key factor in Alzheimer’s disease [130]. While it is intriguing that two 3q29Del locus genes are involved in the process of GPI anchor biosynthesis, it remains unclear whether hemizygosity of both genes would have any deleterious effects in the developing nervous system. However, at least 19 GPI anchor-related genes are known to cause GPI biosynthesis defect disorders which can result in seizures and developmental delay [131] though only when both alleles are mutated. While gnomAD analysis does not indicate either *PIGX* or *PIGZ* are constrained, it is possible that hemizygosity of both genes could disrupt GPI anchor biosynthesis. Further studies would be required to test this possibility.

*Palmitoylation*

**ZDHHC19 (No evidence)**

*ZDHHC19* encodes a protein found in the Golgi membrane, endoplasmic reticulum, and perinucleolar compartment, with high expression in adult testis and minimal to no expression in other tissues [132]. Knockout studies in mice revealed that ZDHHC19 has a role in male fertility. Spermatozoa in knockout mice presented with defects of the head and tail, impacting motility and oocyte fertilization [133]. The available evidence and gnomAD analysis indicate it is unlikely that hemizygosity of *ZDHHC19* would impact neurodevelopment.

**Metabolism**

**SLC51A (No evidence)**

*SLC51A* (aka *OSTA*) encodes a protein involved in intestinal transport of bile acids (aka OST-α). SLC51A also has the ability to transport steroids through the hepatic system [134]. Knockout of mouse homolog *Osta* showed no physical differences from wild-type but signs of disrupted bile biosynthesis were identified [135]. In humans, biallelic deficiency of OST-α results in severe hepatic dysfunction, including congenital diarrhea and fat malabsorption [136, 137]. Public databases indicate that *SLC51A* expression is restricted to the adrenal gland, gut, and liver.

**PCYT1A (Tier 2)**

*PCYT1A* encodes the enzyme CTP:phosphocholine cytidylyltransferase A (CCTa/PCYT1A) [138]. PCYT1A regulates the formation of phosphatidylcholine (PC) via the Kennedy pathway [139] and also maintains PC levels within cellular and nuclear membranes. PCYT1A is expressed in all tissues but is highly concentrated in the liver, kidney, and heart [140]. Biallelic mutations of *PCYT1A* can cause spondylometaphyseal dysplasia with cone-rod dystrophy, a rare disorder that presents with musculoskeletal malformation, delayed growth, and progressive vision loss [141]. Other vision-related disorders have been linked to *PCYT1A,* such as Leber congenital amaurosis (LCA) and retinal dystrophy [142]. *PCYT1A* mutations have also been linked to congenital lipodystrophy and fatty liver disease, which are caused by the disruption of phosphatidylcholine synthesis [143]. Complete knockout of *Pcyt1a* in mice resulted in embryonic lethality by day 3.5 and a failure to implant. Heterozygous adult mice had decreased levels of Pcyt1a in the liver and other tissues, while embryos displayed signs of delayed development at the morula stage [144].

**BDH1 (Tier 2)**

*BDH1* is a member of the short-chain dehydrogenase/reductase gene family and utilizes phosphatidylcholine to initiate enzymatic activity [145], illustrating a potential connection to *PCYT1A* haploinsufficiency. BDH1 is highly expressed in the liver and gut and initiates the catalysis of ketone bodies formed in the liver, and its dysfunction can lead to jaundice [146]. Ketone bodies serve as an alternative fuel source in glucose-dependent tissues, like the brain, when glucose is absent. In mice, Bdh1 assists in the reduction of cardiac stress following an apical cardiac infarction [146], and attenuates diabetes-induced atherosclerosis [147]. In light of recent findings of altered fat metabolism in 3q29Del mice [148] and mitochondrial energy metabolism dysfunction in human 3q29Del cells [49], *BDH1* is a reasonable candidate to contribute as a phenotypic driver gene. In addition, it is notable that BDH1 is the only 3q29Del gene that is listed in the Mitocarta3.0 inventory of mitochondrial proteins [75]. However, gnomAD (v4.1) data does not indicate genic constraint on *BDH1*, suggesting that *BDH1* may interact with other 3q29Del genes to produce metabolic and/or mitochondrial phenotypes.

**Iron Homeostasis**

**TFRC (Tier 1)**

*TFRC* encodes the transferrin receptor (TFRC, aka TFR1), which binds to and internalizes iron-bound transferrin and thereby mediates the primary route for cellular iron import [149]. Homozygous *Tfrc* knockout mice are not viable past embryonic day 12.5, suffering from severe anemia, edema, necrosis, and neurological abnormalities [150]. Heterozygous *Tfrc* knockout animals were reported to be grossly normal but were found to have reduced iron levels in liver and spleen and evidence of iron deficiency in erythrocyte analysis. Barring compensation from another 3q29 locus gene – or chromosome and gene-regulatory differences in the two models – 3q29Del mice could be expected to have similar phenotypes, though this has not been reported. Homozygous *Tfrc* knockout in neural crest cells (*Wnt1^Cre^*) led to severe embryonic craniofacial abnormalities and reduced TGF-β and BMP signaling [151], which could impact neural differentiation and axon specification [152, 153]. Knockdown of *TFRC* in human neural progenitor cells was found to decrease levels of mitochondrial fission-associated proteins DRP1 and FIS1 and increase levels of fusion-associated proteins OPA1 and MFN2 [154]. Furthermore, TFRC was found to interact with GSK3β, another signaling molecule important for neurogenesis, neuronal polarization, and axon growth [155] and levels of GSK3β were found to correlate with TFRC protein levels [154]. Additional experiments will be required to determine how hemizygosity of *TFRC* as in 3q29Del may impact these pathways in developing neural cells.

From a translational perspective, iron deficiency, which is a potential risk for individuals with 3q29Del syndrome due to hemizygosity of *TFRC*, is associated with a similar spectrum of neurodevelopmental syndromes as 3q29Del including significantly elevated risk for schizophrenia [156–158]. The mechanisms by which iron deficiency increases risk for these NDDs in humans are not clear, but studies in mammalian experimental systems have shown that genetic or environmental iron deficiency can impair neuronal metabolism and maturation [159–161]. Further studies are needed to understand if 3q29Del indeed increases neurodevelopmental vulnerability to iron deficiency and if iron deficiency could be an environmental mediator of 3q29Del phenotype severity.

**MELTF (No evidence)**

*MELTF* (aka *MFI2*) encodes the cell-surface expressed, iron-binding glycoprotein melanotransferrin (MTf) [162]. MTf is highly conserved across species and its genetic sequence shares 40% similarity to other iron-binding proteins such as lactoferrin and transferrin [163]. While it is intriguing that a second 3q29 locus gene is potentially involved in iron homeostasis – and could represent a “second hit” to the pathway – it remains unclear whether MTf participates in cellular iron homeostasis, particularly in the brain [164].

**Immune System**

**TM4SF19 (Tier 3)**

Tetraspanins are integral membrane proteins involved in numerous cellular functions, such as proliferation, apoptosis, migration, and signal transduction, while also critical for cell-cell fusion [165]. TM4SF19 is a tetraspanin family protein found to regulate osteoclast function [166]. TM4SF19 has been shown to be a lysosomal membrane protein expressed in macrophages, involved in reduction of inflammation of adipose tissue. *Tm4sf19* null mice displayed altered metabolic function, with KO mice showing improved glucose and insulin tolerance [167]. Interestingly, splice-site and missense variants of *TM4SF19* have been identified in ASD candidate gene research [14, 168], indicating that this poorly-annotated protein deserves further investigation.

**NRROS (Tier 3)**

*NRROS* is a leucine-rich repeat containing transmembrane protein that was named for its function as a negative regulator of reactive oxygen species in immune cells [169]. Complete knockout of *Nrros* resulted in enhanced bactericidal activity by phagocytes but oxidative damage to the CNS [169]. In fact, knockout of *Nrros* resulted in early mortality, developmental delays, and spontaneous neurological disorders in mice [170] and biallelic human mutations were found to cause demyelination, neuronal loss, and astrogliosis [171]. *NRROS* expression is limited to microglia in the parenchyma of the central nervous system and is most critical at embryonic and postnatal stages of development [171]. Homozygous or compound heterozygous mutations of *NRROS* have been linked to early-onset seizure disorders in humans, with brain calcification and neurodegeneration [171, 172]. LOEUF does not indicate that *NRROS* is a constrained gene (Fig. 2) and further experimentation will be required to determine if hemizygosity of *NRROS* as in 3q29Del would contribute to neuropsychiatric phenotypes.

**Ciliary**

**DYNLT2B (Tier 2)**

*DYNLT2B* (aka *TCTEX1D2*) is part of a family of cytoplasmic dyneins responsible for intracellular motility and is specifically necessary for retrograde ciliary transport [173]. Skeletal ciliopathies are commonly associated with mutations in this gene family. DYNLT2B has been linked to Short-Rib Thoracic Dysplasia-17 (SRTD17) with or without polydactyly, a group of autosomal recessive disorders that include Ellis-van Creveld syndrome (EVC), Jeune syndrome, and asphyxiating thoracic dystrophy (ATD). SRTD17 presents with numerous skeletal abnormalities like extra fingers and toes, a bell-shaped chest, shortened ribs and limbs, and an abnormal pelvis. Symptoms also can include renal dysfunction, and pancreatic, hepatic, and retinal abnormalities [174]. ATD is the most severe SRTD17 disorder, causing severe hypoxia that often leads to death in early childhood [175]. Knockdown of *DYNLT2B* in zebrafish resulted in numerous physiological abnormalities, including ventrally curved bodies, hydrocephalus, malformed otoliths, and small eyes [176]. Few embryos survived past four days post fertilization; those that did displayed abnormal craniofacial formation, severe edema, pronephric cysts, and mild developmental delays [176]. LOEUF analysis does not indicate *DYNLT2B* is constrained or a likely phenotypic driver, though it appears to be expressed at very high levels in the developing human brain (Fig. 2, Supp. Fig. 1). It is not known if hemizygosity of this gene in combination with another 3q29 gene, such as *CEP19* (below), could contribute to human neurodevelopmental phenotypes.

**CEP19 (Tier 3)**

*CEP19* is expressed throughout the body and at high levels in the testes. CEP19 is also a ciliary protein, forming a pathway with RABL2 to activate the formation of cilia and is necessary for microtubules to anchor along centrosomes [177]. A human familial study suggested that heterozygous mutation of *CEP19* has no metabolic impact, however homozygous carriers displayed symptoms of metabolic disorder, most commonly Type 2 diabetes, and males had little to no sperm count [178]. *CEP19* homozygous truncating mutation has also been linked to Bardet-Biedl syndrome (BBS), which is a rare disorder resulting in the dysfunction of primary cilia [179]. Symptoms of BBS include obesity, kidney abnormalities, intellectual disability, developmental delays, metabolic disorders, and skeletal malformations of the fingers and toes. Homozygous knockout in mice results in spermatogenic failure as well as morbid obesity, while heterozygous mice displayed no apparent differences compared to wild-type animals [178]. While the connection to glucose metabolism is intriguing given metabolic abnormalities in 3q29Del [148], it is not yet clear how hemizygosity of this gene would contribute to 3q29Del phenotypes.

**RNA Processing**

**NCBP2** **(Tier 1)**

*NCBP2* (aka *CBP20*) encodes a nuclear cap binding protein involved in the process of binding monomethylated caps at the 5’ end of pre-mRNAs, which allows mRNA to bind to ribosomes in the cytoplasm [180, 181]. It is highly expressed in the human brain, particularly during prenatal development (Fig. 2). Cap-binding complexes are conserved from yeast to primates and are required for cell growth and proliferation [182]. NCBP2 works directly with the RNA cap [183], is believed to be recruited and stabilized by NCBP1 [184], and has been found to regulate the binding of NCBP3 [185]. *Ncbp2* homozygous deletion results in embryonic lethality prior to organogenesis in mice, and heterozygosity was found to possibly increase anxiety (International Mouse Phenotyping Consortium; IMPC) [186].

In human cells, NCBP1 knockdown resulted in loss-of-function while silenced NCBP2 was apparently compensated for by NCBP3 [183]. In a Drosophila and Xenopus study, homologous knockdown of *NCBP2* resulted in enhanced phenotypes in combinatorial knockdown of other 3q29 homologs [24], though *Drosophila* are not known to express a homolog of the potential compensatory protein NCBP3. Loss of *NCBP2* could have a profound effect on transcript export, but it is not clear that protein levels are reduced. In at least one study in mouse brain, *Ncbp2* mRNA levels were reduced to match gene copy number but Ncbp2 protein levels were not significantly altered compared to wild type, suggesting a post-transcriptional compensatory mechanism [23].

**Unknown Functions**

*SMCO1, WDR53, and NCBP2AS2* are genes of essentially unknown function with little genomic evidence of mutational constraint or brain expression. *NCBP2AS2* encodes a protein that was found to be induced by hypoxia in tumors and secreted to promote angiogenesis [187], though it is unclear if it has a role in the CNS.

**Discussion**

In the quest to understand the biological basis of neurodevelopmental psychiatric conditions, rare CNVs such as 3q29Del have emerged as promising and important targets for investigation. The high disease risk conferred by 3q29Del suggests that this variant would produce strong, observable biological effects using experimental systems such as mouse models [23, 84] and induced-pluripotent stem cell lines [49]. These observations will lead to a better understanding of the pathogenesis of this genetic disorder and may produce new insights into the underlying biology of conditions such as SCZ and ASD. While 3q29Del syndrome certainly qualifies as a rare genetic disorder, estimates indicate a similar prevalence to Huntington’s disease (1:~20,000) [188] and Rett Syndrome (1:10-20,000 females) [189], which have been much more widely studied. PubMed results for ‘Huntington’s’ (>19,000) and ‘Rett Syndrome’ (>4,800) far surpass those for ‘3q29’ (~250). Thus, there is much more to learn about the natural history and phenotypic expression of 3q29Del syndrome, in addition to molecular neurobiology, which will be essential to designing evidence-based interventions to improve well-being and clinical outcomes for people living with this condition.

The oligogenic nature of CNVs increases the complexity of experimental analysis, but also likely makes CNVs a more natural model of disorders such as SCZ, which are considered to have a polygenic basis [15]. Distinguishing potential driver genes from likely “passengers” (i.e. hemizygous genes that do not contribute to phenotype expression) is an important goal for this line of investigation. The available evidence does not support the concept of a single driver gene in the deletion interval producing all or even most neurodevelopmental phenotypes. Therefore, developing a better appreciation of how deficits of multiple genes may impinge on common cellular pathways or in certain cell types will improve our understanding of 3q29Del Syndrome and the basis for psychiatric disease risk associated with this variant.

We conclude that several potential cellular processes may be disrupted by loss of multiple 3q29 genes including ubiquitination/SUMOylation pathways (*UBXN7, RNF168, FBXO45, SENP5*), immune functions (*NRROS, TM4SF19*), and metabolism (*BDH1, PCYT1A, TFRC*). Multiple “hits” to these pathways could have different effects in certain cell types and at discrete stages of development adding to the complexity and opportunity to discover new neurobiology with relevance to human neurodevelopmental and psychiatric conditions.

To provide insight into the functional imperative for each of 3q29 gene as a foundation for further analysis, we have included gene constraint data from the gnomAD database along with gene expression data from BrainSpan to aid in prioritizing potential driver genes. These analyses, in addition to in-depth literature review of functional annotations have led us to prioritize six genes – *TFRC, UBXN7, FBXO45, PAK2, NCBP2,* and *DLG1* – as likely (Tier 1) drivers of 3q29Del-associated neurodevelopmental phenotypes. This conclusion aligns closely with a genome-wide analysis by Collins and colleagues that applied machine-learning approaches to estimate the probability of haplo- or triplosensitivity [28]. They concluded that these same six genes plus *SENP5* are likely sensitive to haploinsufficiency, though that analysis was not focused specifically on neurodevelopmental disorders. Notably, only two 3q29 genes – *PAK2* and *UBXN7* – were predicted to be triplosensitive, which is consistent with findings that the 3q29 duplication is associated with less severe neurodevelopment outcomes [9, 190].

These analyses, naturally, have limitations. The BrainSpan developmental transcriptome does not provide single-cell resolution, which can obscure the contribution of genes such as *NRROS* and *TM4SF19* that seem to be expressed only in certain cell lineages. Moreover, constraint metrics are somewhat limited by gene-level analysis that does not account for compound haploinsufficiency. For example, neither *BDH1* nor *PCYT1A* show evidence of mutational constraint in gnomAD (v4.1.0), but PCYT1A produces phosphatidylcholine, which is required for BDH1 enzymatic activity. Thus, the combined loss of these genes in 3q29Del could have additive or synergistic effects on this pathway. Indeed, a limitation of our analysis is that gene-gene interactions can be unpredictable from single gene functional annotations.

**Experimental Directions**

Gene functional annotations are continually improving but experimentation will be necessary to resolve mechanisms of multi-gene interactions in specific cell types. For example, compound haploinsufficiency of *UBXN7* and *TFRC* could compromise cellular hypoxia responses in the developing CNS. The balance of proliferation and differentiation in radial glia is regulated in part by hypoxia signaling [191]. Hemizygosity of *TFRC* may lead to cellular iron deficiency, which can initiate hypoxia signaling and stabilization of HIF1α. UBXN7 is involved in regulating levels of HIF1α [40, 41]. How do these or other compound hemizygosities due to 3q29Del alter neural circuit development or function? There is evidence that reduction in *Dlg1* expression specifically alters glutamatergic function in the hippocampal dentate gyrus [118]. Would diminished *Pak2* and *Fbxo45* expression in 3q29Del further disrupt this circuit?

Mouse models of 3q29Del exhibit reduced brain mass [23, 84] and recent human neuroimaging studies have reported striking reductions in cerebellar volume [10, 192]. It is not yet known if specific cell types or circuits are compromised in these phenotypes or if these are global phenomena in 3q29Del. Mutations in *CASK*, which encodes a key binding partner of DLG1 [193], are known to cause profound cerebellar hypoplasia [194]. Alternatively, recent findings of altered neural progenitor metabolism [49] could be extrapolated to a deficit in proliferation and differentiation. The human cerebellum may be particularly vulnerable to a reduction in neural progenitor proliferation capacity due to the exponential expansion of granule cell precursors required for healthy development that far exceeds that of mice [195]. Understanding these mechanisms will require further in vivo and in vitro investigation in multiple experimental systems. Approaches such as CRISPRi may help uncover gene-gene interactions [196], and cell-lineage specific mouse driver lines can illuminate gene contributions in certain cell types.

**Conclusion**

Population-level genomic studies have not yet identified any single gene within the 3q29Del interval as a significant risk factor for SCZ or ASD, which suggests that hemizygosity of multiple genes in the deletion locus is required. Our analyses suggest a model in which haploinsufficiency of multiple 3q29 genes converges on several cellular pathways including ubiquitination, metabolism, and synaptic function. These early cellular perturbations may alter neural progenitor behavior, fate specification, and circuit function, ultimately contributing to the neurodevelopmental and psychiatric phenotypes associated with the deletion.

**Acknowledgments**

The authors are grateful for helpful comments provided by Drs. Anthony-Samuel LaMantia and Jennifer Mulle on a draft of this manuscript.

**Funding declaration**

This work was supported by National Institute of Mental Health project K01MH133970 (R.H.P.).

**Conflict of interest**

The authors have no conflicts of interest to declare related to this manuscript.

**Clinical trial number**

Not applicable.

**References**

1. Glassford MR, Rosenfeld JA, Freedman AA, Zwick ME, Mulle JG, Unique Rare Chromosome Disorder Support Group. Novel features of 3q29 deletion syndrome: Results from the 3q29 registry. Am J Med Genet A. 2016;170A:999–1006.

2. Yilmaz F, Gurusamy U, Mosley TJ, Hallast P, Kim K, Mostovoy Y, et al. High level of complexity and global diversity of the 3q29 locus revealed by optical mapping and long-read sequencing. Genome Med. 2023;15:35.

3. Ballif BC, Theisen A, Coppinger J, Gowans GC, Hersh JH, Madan-Khetarpal S, et al. Expanding the clinical phenotype of the 3q29 microdeletion syndrome and characterization of the reciprocal microduplication. Mol Cytogenet. 2008;1:8.

4. Willatt L, Cox J, Barber J, Cabanas ED, Collins A, Donnai D, et al. 3q29 Microdeletion Syndrome: Clinical and Molecular Characterization of a New Syndrome. Am J Hum Genet. 2005;77:154–160.

5. Marshall CR, Howrigan DP, Merico D, Thiruvahindrapuram B, Wu W, Greer DS, et al. Contribution of copy number variants to schizophrenia from a genome-wide study of 41,321 subjects. Nat Genet. 2017;49:27–35.

6. Singh T, Poterba T, Curtis D, Akil H, Al Eissa M, Barchas JD, et al. Rare coding variants in ten genes confer substantial risk for schizophrenia. Nature. 2022;604:509–516.

7. Wawrzonek AJ, Sharp W, Burrell TL, Gillespie SE, Pollak RM, Murphy MM, et al. Symptoms of Pediatric Feeding Disorders Among Individuals with 3q29 Deletion Syndrome: A Case-Control Study. J Dev Behav Pediatr JDBP. 2022;43:e170–e178.

8. Sefik E, Guest RM, Aberizk K, Espana R, Goines K, Novacek DM, et al. Psychosis spectrum symptoms among individuals with schizophrenia-associated copy number variants and evidence of cerebellar correlates of symptom severity. Psychiatry Res. 2024;335:115867.

9. Pollak RM, Zinsmeister MC, Murphy MM, Zwick ME, Emory 3q29 Project, Mulle JG. New phenotypes associated with 3q29 duplication syndrome: Results from the 3q29 registry. Am J Med Genet A. 2020;182:1152–1166.

10. Sanchez Russo R, Gambello MJ, Murphy MM, Aberizk K, Black E, Burrell TL, et al. Deep phenotyping in 3q29 deletion syndrome: recommendations for clinical care. Genet Med Off J Am Coll Med Genet. 2021;23:872–880.

11. LaMantia A-S. Why Does the Face Predict the Brain? Neural Crest Induction, Craniofacial Morphogenesis, and Neural Circuit Development. Front Physiol. 2020;11:610970.

12. Wang Y, Zeng C, Li J, Zhou Z, Ju X, Xia S, et al. PAK2 Haploinsufficiency Results in Synaptic Cytoskeleton Impairment and Autism-Related Behavior. Cell Rep. 2018;24:2029–2041.

13. Li J, Shi M, Ma Z, Zhao S, Euskirchen G, Ziskin J, et al. Integrated systems analysis reveals a molecular network underlying autism spectrum disorders. Mol Syst Biol. 2014;10:774.

14. Iossifov I, Ronemus M, Levy D, Wang Z, Hakker I, Rosenbaum J, et al. De Novo Gene Disruptions in Children on the Autistic Spectrum. Neuron. 2012;74:285–299.

15. LaMantia A-S. Polygenicity in a box: Copy number variants, neural circuit development, and neurodevelopmental disorders. Curr Opin Neurobiol. 2024;89:102917.

16. Iyer J, Girirajan S. Gene discovery and functional assessment of rare copy-number variants in neurodevelopmental disorders. Brief Funct Genomics. 2015;14:315–328.

17. Durand CM, Betancur C, Boeckers TM, Bockmann J, Chaste P, Fauchereau F, et al. Mutations in the gene encoding the synaptic scaffolding protein SHANK3 are associated with autism spectrum disorders. Nat Genet. 2007;39:25–27.

18. Moessner R, Marshall CR, Sutcliffe JS, Skaug J, Pinto D, Vincent J, et al. Contribution of SHANK3 mutations to autism spectrum disorder. Am J Hum Genet. 2007;81:1289–1297.

19. Davenport CM, Teubner BJW, Han SB, Patton MH, Eom T-Y, Garic D, et al. Innate frequency-discrimination hyperacuity in Williams-Beuren syndrome mice. Cell. 2022;185:3877-3895.e21.

20. Curran ME, Atkinson DL, Ewart AK, Morris CA, Leppert MF, Keating MT. The elastin gene is disrupted by a translocation associated with supravalvular aortic stenosis. Cell. 1993;73:159–168.

21. Qiu Y, Arbogast T, Lorenzo SM, Li H, Tang SC, Richardson E, et al. Oligogenic Effects of 16p11.2 Copy-Number Variation on Craniofacial Development. Cell Rep. 2019;28:3320-3328.e4.

22. Kretz PF, Wagner C, Mikhaleva A, Montillot C, Hugel S, Morella I, et al. Dissecting the autism-associated 16p11.2 locus identifies multiple drivers in neuroanatomical phenotypes and unveils a male-specific role for the major vault protein. Genome Biol. 2023;24:261.

23. Rutkowski TP, Purcell RH, Pollak RM, Grewenow SM, Gafford GM, Malone T, et al. Behavioral changes and growth deficits in a CRISPR engineered mouse model of the schizophrenia-associated 3q29 deletion. Mol Psychiatry. 2019;26:772–783.

24. Singh MD, Jensen M, Lasser M, Huber E, Yusuff T, Pizzo L, et al. NCBP2 modulates neurodevelopmental defects of the 3q29 deletion in Drosophila and Xenopus laevis models. PLoS Genet. 2020;16:e1008590.

25. Sefik E, Purcell RH, Emory 3q29 Project, Walker EF, Bassell GJ, Mulle JG. Convergent and distributed effects of the 3q29 deletion on the human neural transcriptome. Transl Psychiatry. 2021;11:357.

26. Gudmundsson S, Singer-Berk M, Watts NA, Phu W, Goodrich JK, Solomonson M, et al. Variant interpretation using population databases: Lessons from gnomAD. Hum Mutat. 2022;43:1012–1030.

27. Chen S, Francioli LC, Goodrich JK, Collins RL, Kanai M, Wang Q, et al. A genomic mutational constraint map using variation in 76,156 human genomes. Nature. 2024;625:92–100.

28. Collins RL, Glessner JT, Porcu E, Lepamets M, Brandon R, Lauricella C, et al. A cross-disorder dosage sensitivity map of the human genome. Cell. 2022;185:3041-3055.e25.

29. Motahari Z, Moody SA, Maynard TM, LaMantia A-S. In the line-up: deleted genes associated with DiGeorge/22q11.2 deletion syndrome: are they all suspects? J Neurodev Disord. 2019;11:7.

30. Kerscher O, Felberbaum R, Hochstrasser M. Modification of proteins by ubiquitin and ubiquitin-like proteins. Annu Rev Cell Dev Biol. 2006;22:159–180.

31. Kawabe H, Brose N. The role of ubiquitylation in nerve cell development. Nat Rev Neurosci. 2011;12:251–268.

32. Bundey S, Hardy C, Vickers S, Kilpatrick MW, Corbett JA. Duplication of the 15q11-13 region in a patient with autism, epilepsy and ataxia. Dev Med Child Neurol. 1994;36:736–742.

33. Burnside RD, Pasion R, Mikhail FM, Carroll AJ, Robin NH, Youngs EL, et al. Microdeletion/microduplication of proximal 15q11.2 between BP1 and BP2: a susceptibility region for neurological dysfunction including developmental and language delay. Hum Genet. 2011;130:517–528.

34. Lopez SJ, Segal DJ, LaSalle JM. UBE3A: An E3 Ubiquitin Ligase With Genome-Wide Impact in Neurodevelopmental Disease. Front Mol Neurosci. 2018;11:476.

35. Yashiro K, Riday TT, Condon KH, Roberts AC, Bernardo DR, Prakash R, et al. Ube3a is required for experience-dependent maturation of the neocortex. Nat Neurosci. 2009;12:777–783.

36. Kong A, Frigge ML, Masson G, Besenbacher S, Sulem P, Magnusson G, et al. Rate of de novo mutations and the importance of father’s age to disease risk. Nature. 2012;488:471–475.

37. O’Roak BJ, Vives L, Girirajan S, Karakoc E, Krumm N, Coe BP, et al. Sporadic autism exomes reveal a highly interconnected protein network of de novo mutations. Nature. 2012;485:246–250.

38. Schuberth C, Buchberger A. UBX domain proteins: major regulators of the AAA ATPase Cdc48/p97. Cell Mol Life Sci CMLS. 2008;65:2360–2371.

39. Braxton JR, Southworth DR. Structural insights of the p97/VCP AAA+ ATPase: How adapter interactions coordinate diverse cellular functionality. J Biol Chem. 2023;299.

40. Yee Koh M, Spivak-Kroizman TR, Powis G. HIF-1 regulation: not so easy come, easy go. Trends Biochem Sci. 2008;33:526–534.

41. Alexandru G, Graumann J, Smith GT, Kolawa NJ, Fang R, Deshaies RJ. UBXD7 binds multiple ubiquitin ligases and implicates p97 in HIF1alpha turnover. Cell. 2008;134:804–816.

42. Bandau S, Knebel A, Gage ZO, Wood NT, Alexandru G. UBXN7 docks on neddylated cullin complexes using its UIM motif and causes HIF1α accumulation. BMC Biol. 2012;10:36.

43. Tebay LE, Robertson H, Durant ST, Vitale SR, Penning TM, Dinkova-Kostova AT, et al. Mechanisms of activation of the transcription factor Nrf2 by redox stressors, nutrient cues, and energy status and the pathways through which it attenuates degenerative disease. Free Radic Biol Med. 2015;88:108–146.

44. Maxwell PH, Wiesener MS, Chang GW, Clifford SC, Vaux EC, Cockman ME, et al. The tumour suppressor protein VHL targets hypoxia-inducible factors for oxygen-dependent proteolysis. Nature. 1999;399:271–275.

45. Tao S, Liu P, Luo G, Rojo de la Vega M, Chen H, Wu T, et al. p97 Negatively Regulates NRF2 by Extracting Ubiquitylated NRF2 from the KEAP1-CUL3 E3 Complex. Mol Cell Biol. 2017;37:e00660-16.

46. Di Gregorio J, Cilenti L, Ambivero CT, Andl T, Liao R, Zervos AS. UBXN7 cofactor of CRL3KEAP1 and CRL2VHL ubiquitin ligase complexes mediates reciprocal regulation of NRF2 and HIF-1α proteins. Biochim Biophys Acta Mol Cell Res. 2021;1868:118963.

47. Peng J, Ren K-D, Yang J, Luo X-J. Mitochondrial E3 ubiquitin ligase 1: A key enzyme in regulation of mitochondrial dynamics and functions. Mitochondrion. 2016;28:49–53.

48. Cilenti L, Di Gregorio J, Ambivero CT, Andl T, Liao R, Zervos AS. Mitochondrial MUL1 E3 ubiquitin ligase regulates Hypoxia Inducible Factor (HIF-1α) and metabolic reprogramming by modulating the UBXN7 cofactor protein. Sci Rep. 2020;10:1609.

49. Purcell RH, Sefik E, Werner E, King AT, Mosley TJ, Merritt-Garza ME, et al. Cross-species analysis identifies mitochondrial dysregulation as a functional consequence of the schizophrenia-associated 3q29 deletion. Sci Adv. 2023;9.

50. Oh E, Mark KG, Mocciaro A, Watson ER, Prabu JR, Cha DD, et al. Gene expression and cell identity controlled by anaphase-promoting complex. Nature. 2020;579:136–140.

51. Kang Y, Zhou Y, Li Y, Han Y, Xu J, Niu W, et al. A human forebrain organoid model of fragile X syndrome exhibits altered neurogenesis and highlights new treatment strategies. Nat Neurosci. 2021;24:1377–1391.

52. Urresti J, Zhang P, Moran-Losada P, Yu N-K, Negraes PD, Trujillo CA, et al. Cortical organoids model early brain development disrupted by 16p11.2 copy number variants in autism. Mol Psychiatry. 2021;26:7560–7580.

53. Data Download :: BrainSpan: Atlas of the Developing Human Brain. https://www.brainspan.org/static/download.html. Accessed 16 March 2026.

54. Doil C, Mailand N, Bekker-Jensen S, Menard P, Larsen DH, Pepperkok R, et al. RNF168 binds and amplifies ubiquitin conjugates on damaged chromosomes to allow accumulation of repair proteins. Cell. 2009;136:435–446.

55. Pinato S, Scandiuzzi C, Arnaudo N, Citterio E, Gaudino G, Penengo L. RNF168, a new RING finger, MIU-containing protein that modifies chromatin by ubiquitination of histones H2A and H2AX. BMC Mol Biol. 2009;10:55.

56. Stewart GS, Panier S, Townsend K, Al-Hakim AK, Kolas NK, Miller ES, et al. The RIDDLE Syndrome Protein Mediates a Ubiquitin-Dependent Signaling Cascade at Sites of DNA Damage. Cell. 2009;136:420–434.

57. Acs K, Luijsterburg MS, Ackermann L, Salomons FA, Hoppe T, Dantuma NP. The AAA-ATPase VCP/p97 promotes 53BP1 recruitment by removing L3MBTL1 from DNA double-strand breaks. Nat Struct Mol Biol. 2011;18:1345–1350.

58. Stewart GS, Stankovic T, Byrd PJ, Wechsler T, Miller ES, Huissoon A, et al. RIDDLE immunodeficiency syndrome is linked to defects in 53BP1-mediated DNA damage signaling. Proc Natl Acad Sci U S A. 2007;104:16910–16915.

59. Gu X, Hou Y, Chen Y, Ou R, Cao B, Wei Q, et al. Enrichment of rare variants in E3 ubiquitin ligase genes in Early onset Parkinson’s disease. Neurobiol Aging. 2022;109:273–278.

60. Kong L, Pokatayev V, Lefkovith A, Carter GT, Creasey EA, Krishna C, et al. The landscape of immune dysregulation in Crohn’s disease revealed through single-cell transcriptomic profiling in the ileum and colon. Immunity. 2023;56:444-458.e5.

61. Yoshida K. Characterization of estrogen-induced F-box protein FBXO45. Oncol Rep. 2005;14:531–535.

62. Saiga T, Fukuda T, Matsumoto M, Tada H, Okano HJ, Okano H, et al. Fbxo45 forms a novel ubiquitin ligase complex and is required for neuronal development. Mol Cell Biol. 2009;29:3529–3543.

63. Peschiaroli A, Scialpi F, Bernassola F, Pagano M, Melino G. The F-box protein FBXO45 promotes the proteasome-dependent degradation of p73. Oncogene. 2009;28:3157–3166.

64. Tada H, Okano HJ, Takagi H, Shibata S, Yao I, Matsumoto M, et al. Fbxo45, a Novel Ubiquitin Ligase, Regulates Synaptic Activity2. J Biol Chem. 2010;285:3840–3849.

65. Na Y, Calvo-Jiménez E, Kon E, Cao H, Jossin Y, Cooper JA. Fbxo45 Binds SPRY Motifs in the Extracellular Domain of N-Cadherin and Regulates Neuron Migration during Brain Development. Mol Cell Biol. 2020;40:e00539-19.

66. Murthy V, Han S, Beauchamp RL, Smith N, Haddad LA, Ito N, et al. Pam and its ortholog highwire interact with and may negatively regulate the TSC1.TSC2 complex. J Biol Chem. 2004;279:1351–1358.

67. Bahl S, Chiang C, Beauchamp RL, Neale BM, Daly MJ, Gusella JF, et al. Lack of association of rare functional variants in TSC1/TSC2 genes with autism spectrum disorder. Mol Autism. 2013;4:5.

68. Wang C, Koide T, Kimura H, Kunimoto S, Yoshimi A, Nakamura Y, et al. Novel rare variants in F-box protein 45 (FBXO45) in schizophrenia. Schizophr Res. 2014;157:149–156.

69. Shimanoe C, Hachiya T, Hara M, Nishida Y, Tanaka K, Sutoh Y, et al. A genome-wide association study of coping behaviors suggests FBXO45 is associated with emotional expression. Genes Brain Behav. 2019;18:e12481.

70. Di Bacco A, Ouyang J, Lee H-Y, Catic A, Ploegh H, Gill G. The SUMO-specific protease SENP5 is required for cell division. Mol Cell Biol. 2006;26:4489–4498.

71. Gong L, Yeh ETH. Characterization of a family of nucleolar SUMO-specific proteases with preference for SUMO-2 or SUMO-3. J Biol Chem. 2006;281:15869–15877.

72. Bhagwat NR, Owens SN, Ito M, Boinapalli JV, Poa P, Ditzel A, et al. SUMO is a pervasive regulator of meiosis. eLife. 2021;10:e57720.

73. Li X-C, Zeng Y, Sun R-R, Liu M, Chen S, Zhang P-Y. SUMOylation in cardiac disorders - a review. Eur Rev Med Pharmacol Sci. 2017;21:1583–1587.

74. Zunino R, Braschi E, Xu L, McBride HM. Translocation of SenP5 from the nucleoli to the mitochondria modulates DRP1-dependent fission during mitosis. J Biol Chem. 2009;284:17783–17795.

75. Rath S, Sharma R, Gupta R, Ast T, Chan C, Durham TJ, et al. MitoCarta3.0: an updated mitochondrial proteome now with sub-organelle localization and pathway annotations. Nucleic Acids Res. 2021;49:D1541–D1547.

76. Frank S, Gaume B, Bergmann-Leitner ES, Leitner WW, Robert EG, Catez F, et al. The role of dynamin-related protein 1, a mediator of mitochondrial fission, in apoptosis. Dev Cell. 2001;1:515–525.

77. Prudent J, Zunino R, Sugiura A, Mattie S, Shore GC, McBride HM. MAPL SUMOylation of Drp1 Stabilizes an ER/Mitochondrial Platform Required for Cell Death. Mol Cell. 2015;59:941–955.

78. Akiyama H, Nakadate K, Sakakibara S-I. Synaptic localization of the SUMOylation-regulating protease SENP5 in the adult mouse brain. J Comp Neurol. 2018;526:990–1005.

79. Yamada S, Sato A, Ishihara N, Akiyama H, Sakakibara S. Drp1 SUMO/deSUMOylation by Senp5 isoforms influences ER tubulation and mitochondrial dynamics to regulate brain development. iScience. 2021;24:103484.

80. Rudel T, Bokoch GM. Membrane and morphological changes in apoptotic cells regulated by caspase-mediated activation of PAK2. Science. 1997;276:1571–1574.

81. Marlin JW, Chang Y-WE, Ober M, Handy A, Xu W, Jakobi R. Functional PAK-2 knockout and replacement with a caspase cleavage-deficient mutant in mice reveals differential requirements of full-length PAK-2 and caspase-activated PAK-2p34. Mamm Genome. 2011;22:306–317.

82. Bokoch GM. Biology of the p21-activated kinases. Annu Rev Biochem. 2003;72:743–781.

83. Alves MM, Fuhler GM, Queiroz KCS, Scholma J, Goorden S, Anink J, et al. PAK2 is an effector of TSC1/2 signaling independent of mTOR and a potential therapeutic target for Tuberous Sclerosis Complex. Sci Rep. 2015;5:14534.

84. Baba M, Yokoyama K, Seiriki K, Naka Y, Matsumura K, Kondo M, et al. Psychiatric-disorder-related behavioral phenotypes and cortical hyperactivity in a mouse model of 3q29 deletion syndrome. Neuropsychopharmacol Off Publ Am Coll Neuropsychopharmacol. 2019;44:2125–2135.

85. Misra UK, Deedwania R, Pizzo SV. Binding of activated alpha2-macroglobulin to its cell surface receptor GRP78 in 1-LN prostate cancer cells regulates PAK-2-dependent activation of LIMK. J Biol Chem. 2005;280:26278–26286.

86. Cheng T-Y, Yang Y-C, Wang H-P, Tien Y-W, Shun C-T, Huang H-Y, et al. Pyruvate kinase M2 promotes pancreatic ductal adenocarcinoma invasion and metastasis through phosphorylation and stabilization of PAK2 protein. Oncogene. 2018;37:1730–1742.

87. Banko MR, Allen JJ, Schaffer BE, Wilker EW, Tsou P, White JL, et al. Chemical genetic screen for AMPKα2 substrates uncovers a network of proteins involved in mitosis. Mol Cell. 2011;44:878–892.

88. Kong D, Dagon Y, Campbell JN, Guo Y, Yang Z, Yi X, et al. A Postsynaptic AMPK→p21-Activated Kinase Pathway Drives Fasting-Induced Synaptic Plasticity in AgRP Neurons. Neuron. 2016;91:25–33.

89. Kořánová T, Dvořáček L, Grebeňová D, Röselová P, Obr A, Kuželová K. PAK1 and PAK2 in cell metabolism regulation. J Cell Biochem. 2022;123:375–389.

90. Gupta A, Ajith A, Singh S, Panday RK, Samaiya A, Shukla S. PAK2-c-Myc-PKM2 axis plays an essential role in head and neck oncogenesis via regulating Warburg effect. Cell Death Dis. 2018;9:825.

91. Wang Y, Zhang K, Guo J, Yang S, Shi X, Pan J, et al. Loss-of-Function of p21-Activated Kinase 2 Links BMP Signaling to Neural Tube Patterning Defects. Adv Sci Weinh Baden-Wurtt Ger. 2023;10:e2204018.

92. Hu B, Moiseev D, Schena I, Faezov B, Dunbrack R, Chernoff J, et al. PAK2 is necessary for myelination in the peripheral nervous system. Brain J Neurol. 2024;147:1809–1821.

93. Schnur RE, Dvořáček L, Kalsner L, Shapiro FL, Grebeňová D, Yanni D, et al. New kinase-deficient PAK2 variants associated with Knobloch syndrome type 2. Clin Genet. 2024;106.

94. Yahia A, Li D, Lejerkrans S, Rajagopalan S, Kalnak N, Tammimies K. Whole exome sequencing and polygenic assessment of a Swedish cohort with severe developmental language disorder. Hum Genet. 2024;143:169–183.

95. Taglieri DM, Ushio-Fukai M, Monasky MM. P21-activated kinase in inflammatory and cardiovascular disease. Cell Signal. 2014;26:2060–2069.

96. Rejeb I, Saillour Y, Castelnau L, Julien C, Bienvenu T, Taga P, et al. A novel splice mutation in PAK3 gene underlying mental retardation with neuropsychiatric features. Eur J Hum Genet. 2008;16:1358–1363.

97. Marziali F, Dizanzo MP, Cavatorta AL, Gardiol D. Differential expression of DLG1 as a common trait in different human diseases: an encouraging issue in molecular pathology. Biol Chem. 2019;400:699–710.

98. Lambert JT, Hill TC, Park DK, Culp JH, Zito K. Protracted and asynchronous accumulation of PSD95-family MAGUKs during maturation of nascent dendritic spines. Dev Neurobiol. 2017;77:1161–1174.

99. Nooh MM, Chumpia MM, Hamilton TB, Bahouth SW. Sorting of β1-Adrenergic Receptors Is Mediated by Pathways That Are Either Dependent on or Independent of Type I PDZ, Protein Kinase A (PKA), and SAP97. J Biol Chem. 2014;289:2277–2294.

100. Stephenson JR, Paavola KJ, Schaefer SA, Kaur B, Van Meir EG, Hall RA. Brain-specific angiogenesis inhibitor-1 signaling, regulation, and enrichment in the postsynaptic density. J Biol Chem. 2013;288:22248–22256.

101. Stephenson JR, Purcell RH, Hall RA. The BAI subfamily of adhesion GPCRs: synaptic regulation and beyond. Trends Pharmacol Sci. 2014;35:208–215.

102. Sakurai T, Kamakura S, Hayase J, Kohda A, Nakamura M, Sumimoto H. GPR125 (ADGRA3) is an autocleavable adhesion GPCR that traffics with Dlg1 to the basolateral membrane and regulates epithelial apicobasal polarity. J Biol Chem. 2022;298:102475.

103. Hilbig D, Sittig D, Hoffmann F, Rothemund S, Warmt E, Quaas M, et al. Mechano-Dependent Phosphorylation of the PDZ-Binding Motif of CD97/ADGRE5 Modulates Cellular Detachment. Cell Rep. 2018;24:1986–1995.

104. Dunn HA, Walther C, Yuan GY, Caetano FA, Godin CM, Ferguson SSG. Role of SAP97 in the regulation of 5-HT2AR endocytosis and signaling. Mol Pharmacol. 2014;86:275–283.

105. Dunn HA, Walther C, Godin CM, Hall RA, Ferguson SSG. Role of SAP97 protein in the regulation of corticotropin-releasing factor receptor 1 endocytosis and extracellular signal-regulated kinase 1/2 signaling. J Biol Chem. 2013;288:15023–15034.

106. Cho C, Wang Y, Smallwood PM, Williams J, Nathans J. Dlg1 activates beta-catenin signaling to regulate retinal angiogenesis and the blood-retina and blood-brain barriers. eLife. 2019;8:e45542.

107. Li D, Specht CG, Waites CL, Butler-Munro C, Leal-Ortiz S, Foote JW, et al. SAP97 directs NMDA receptor spine targeting and synaptic plasticity. J Physiol. 2011;589:4491–4510.

108. Gardoni F, Mauceri D, Fiorentini C, Bellone C, Missale C, Cattabeni F, et al. CaMKII-dependent phosphorylation regulates SAP97/NR2A interaction. J Biol Chem. 2003;278:44745–44752.

109. Underhill SM, Wheeler DS, Amara SG. Differential regulation of two isoforms of the glial glutamate transporter EAAT2 by DLG1 and CaMKII. J Neurosci Off J Soc Neurosci. 2015;35:5260–5270.

110. Leonard AS, Davare MA, Horne MC, Garner CC, Hell JW. SAP97 is associated with the alpha-amino-3-hydroxy-5-methylisoxazole-4-propionic acid receptor GluR1 subunit. J Biol Chem. 1998;273:19518–19524.

111. Sans N, Racca C, Petralia RS, Wang YX, McCallum J, Wenthold RJ. Synapse-associated protein 97 selectively associates with a subset of AMPA receptors early in their biosynthetic pathway. J Neurosci Off J Soc Neurosci. 2001;21:7506–7516.

112. Jeyifous O, Waites CL, Specht CG, Fujisawa S, Schubert M, Lin EI, et al. SAP97 and CASK mediate sorting of NMDA receptors through a previously unknown secretory pathway. Nat Neurosci. 2009;12:1011–1019.

113. Lin EI, Jeyifous O, Green WN. CASK regulates SAP97 conformation and its interactions with AMPA and NMDA receptors. J Neurosci Off J Soc Neurosci. 2013;33:12067–12076.

114. Leonoudakis D, Mailliard W, Wingerd K, Clegg D, Vandenberg C. Inward rectifier potassium channel Kir2.2 is associated with synapse-associated protein SAP97. J Cell Sci. 2001;114:987–998.

115. Gardoni F, Mauceri D, Marcello E, Sala C, Di Luca M, Jeromin A. SAP97 directs the localization of Kv4.2 to spines in hippocampal neurons: regulation by CaMKII. J Biol Chem. 2007;282:28691–28699.

116. Regalado MP, Terry-Lorenzo RT, Waites CL, Garner CC, Malenka RC. Transsynaptic signaling by postsynaptic synapse-associated protein 97. J Neurosci Off J Soc Neurosci. 2006;26:2343–2357.

117. Cotter L, Ozçelik M, Jacob C, Pereira JA, Locher V, Baumann R, et al. Dlg1-PTEN interaction regulates myelin thickness to prevent damaging peripheral nerve overmyelination. Science. 2010;328:1415–1418.

118. Kay Y, Tsan L, Davis EA, Tian C, Décarie-Spain L, Sadybekov A, et al. Schizophrenia-associated SAP97 mutations increase glutamatergic synapse strength in the dentate gyrus and impair contextual episodic memory in rats. Nat Commun. 2022;13:798.

119. Grice SJ, Liu J-L, Webber C. Synergistic interactions between Drosophila orthologues of genes spanned by de novo human CNVs support multiple-hit models of autism. PLoS Genet. 2015;11:e1004998.

120. Kirov G, Pocklington AJ, Holmans P, Ivanov D, Ikeda M, Ruderfer D, et al. De novo CNV analysis implicates specific abnormalities of postsynaptic signalling complexes in the pathogenesis of schizophrenia. Mol Psychiatry. 2012;17:142–153.

121. Trubetskoy V, Pardiñas AF, Qi T, Panagiotaropoulou G, Awasthi S, Bigdeli TB, et al. Mapping genomic loci implicates genes and synaptic biology in schizophrenia. Nature. 2022;604:502–508.

122. Fromer M, Pocklington AJ, Kavanagh DH, Williams HJ, Dwyer S, Gormley P, et al. De novo mutations in schizophrenia implicate synaptic networks. Nature. 2014;506:179–184.

123. Sato J, Shimazu D, Yamamoto N, Nishikawa T. An association analysis of synapse-associated protein 97 (SAP97) gene in schizophrenia. J Neural Transm Vienna Austria 1996. 2008;115:1355–1365.

124. Uezato A, Kimura-Sato J, Yamamoto N, Iijima Y, Kunugi H, Nishikawa T. Further evidence for a male-selective genetic association of synapse-associated protein 97 (SAP97) gene with schizophrenia. Behav Brain Funct BBF. 2012;8:2.

125. Uezato A, Yamamoto N, Jitoku D, Haramo E, Hiraaki E, Iwayama Y, et al. Genetic and molecular risk factors within the newly identified primate-specific exon of the SAP97/DLG1 gene in the 3q29 schizophrenia-associated locus. Am J Med Genet Part B Neuropsychiatr Genet Off Publ Int Soc Psychiatr Genet. 2017;174:798–807.

126. Xu X, Wang Y, Zhou X, Yin J, Yu H, Wen X, et al. The genetic variations in SAP97 gene and the risk of schizophrenia in the Chinese Han population: a further study. Psychiatr Genet. 2020;30:110–118.

127. Ashida H, Hong Y, Murakami Y, Shishioh N, Sugimoto N, Kim YU, et al. Mammalian PIG-X and Yeast Pbn1p Are the Essential Components of Glycosylphosphatidylinositol-Mannosyltransferase I. Mol Biol Cell. 2005;16:1439–1448.

128. Torres-Valdetano Á, Vallejo-Ruiz V, Milflores-Flores L, Martínez-Morales P. Role of PIGM and PIGX in glycosylphosphatidylinositol biosynthesis and human health (Review). Biomed Rep. 2024;20:57.

129. Taron BW, Colussi PA, Wiedman JM, Orlean P, Taron CH. Human Smp3p Adds a Fourth Mannose to Yeast and Human Glycosylphosphatidylinositol Precursors *in Vivo**. J Biol Chem. 2004;279:36083–36092.

130. Toyn JH, Lin X-A, Thompson MW, Guss V, Meredith JE, Sankaranarayanan S, et al. Viable mouse gene ablations that robustly alter brain Aβ levels are rare. BMC Neurosci. 2010;11:143.

131. Wu T, Yin F, Guang S, He F, Yang L, Peng J. The Glycosylphosphatidylinositol biosynthesis pathway in human diseases. Orphanet J Rare Dis. 2020;15:129.

132. Wu Y, Zhang X, Zhang X, Liu S, Zhang J, Sun S, et al. ZDHHC19 localizes to the cell membrane of spermatids and is involved in spermatogenesis†. Biol Reprod. 2022;106:477–486.

133. Wang S, Qiao H, Wang P, Wang Y, Qin D. ZDHHC19 Is Dispensable for Spermatogenesis, but Is Essential for Sperm Functions in Mice. Int J Mol Sci. 2021;22:8894.

134. Ballatori N, Christian WV, Lee JY, Dawson PA, Soroka CJ, Boyer JL, et al. OSTalpha-OSTbeta: a major basolateral bile acid and steroid transporter in human intestinal, renal, and biliary epithelia. Hepatol Baltim Md. 2005;42:1270–1279.

135. Rao A, Haywood J, Craddock AL, Belinsky MG, Kruh GD, Dawson PA. The organic solute transporter α-β, Ostα-Ostβ, is essential for intestinal bile acid transport and homeostasis. Proc Natl Acad Sci. 2008;105:3891–3896.

136. Gao E, Cheema H, Waheed N, Mushtaq I, Erden N, Nelson‐Williams C, et al. Organic Solute Transporter Alpha Deficiency: A Disorder With Cholestasis, Liver Fibrosis, and Congenital Diarrhea. Hepatology. 2020;71:1879.

137. Tronstad RR, Berland S, Tjora E, El Jellas K, Aukrust I, Kristensen K, et al. Fat Malabsorption and Ursodeoxycholic Acid Treatment in Children With Reduced Organic Solute Transporter-α (SLC51A) Expression. JPGN Rep. 2022;3:e229.

138. Haider A, Wei Y-C, Lim K, Barbosa AD, Liu C-H, Weber U, et al. PCYT1A Regulates Phosphatidylcholine Homeostasis from the Inner Nuclear Membrane in Response to Membrane Stored Curvature Elastic Stress. Dev Cell. 2018;45:481-495.e8.

139. Tavasoli M, Lahire S, Reid T, Brodovsky M, McMaster CR. Genetic diseases of the Kennedy pathways for membrane synthesis. J Biol Chem. 2020;295:17877–17886.

140. Karim M, Jackson P, Jackowski S. Gene structure, expression and identification of a new CTP:phosphocholine cytidylyltransferase β isoform. Biochim Biophys Acta BBA - Mol Cell Biol Lipids. 2003;1633:1–12.

141. Yamamoto GL, Baratela WAR, Almeida TF, Lazar M, Afonso CL, Oyamada MK, et al. Mutations in *PCYT1A* Cause Spondylometaphyseal Dysplasia with Cone-Rod Dystrophy. Am J Hum Genet. 2014;94:113–119.

142. Testa F, Filippelli M, Brunetti-Pierri R, Di Fruscio G, Di Iorio V, Pizzo M, et al. Mutations in the PCYT1A gene are responsible for isolated forms of retinal dystrophy. Eur J Hum Genet. 2017;25:651–655.

143. Payne F, Lim K, Girousse A, Brown RJ, Kory N, Robbins A, et al. Mutations disrupting the Kennedy phosphatidylcholine pathway in humans with congenital lipodystrophy and fatty liver disease. Proc Natl Acad Sci U S A. 2014;111:8901–8906.

144. Wang L, Magdaleno S, Tabas I, Jackowski S. Early Embryonic Lethality in Mice with Targeted Deletion of the CTP:Phosphocholine Cytidylyltransferase α Gene (Pcyt1a). Mol Cell Biol. 2005;25:3357–3363.

145. Marks AR, McIntyre JO, Duncan TM, Erdjument-Bromage H, Tempst P, Fleischer S. Molecular cloning and characterization of (R)-3-hydroxybutyrate dehydrogenase from human heart. J Biol Chem. 1992;267:15459–15463.

146. Horton JL, Davidson MT, Kurishima C, Vega RB, Powers JC, Matsuura TR, et al. The failing heart utilizes 3-hydroxybutyrate as a metabolic stress defense. JCI Insight. 2019;4:124079.

147. Lin J, Ren Q, Zhang F, Gui J, Xiang X, Wan Q. D-β-Hydroxybutyrate Dehydrogenase Mitigates Diabetes-Induced Atherosclerosis through the Activation of Nrf2. Thromb Haemost. 2023;123:1003–1015.

148. Pollak RM, Purcell RH, Rutkowski TP, Malone T, Pachura KJ, Bassell GJ, et al. Metabolic effects of the schizophrenia-associated 3q29 deletion. Transl Psychiatry. 2022;12:66.

149. Cheng Y, Zak O, Aisen P, Harrison SC, Walz T. Structure of the human transferrin receptor-transferrin complex. Cell. 2004;116:565–576.

150. Levy JE, Jin O, Fujiwara Y, Kuo F, Andrews NC. Transferrin receptor is necessary for development of erythrocytes and the nervous system. Nat Genet. 1999;21:396–399.

151. Lei R, Zhang K, Liu K, Shao X, Ding Z, Wang F, et al. Transferrin receptor facilitates TGF-β and BMP signaling activation to control craniofacial morphogenesis. Cell Death Dis. 2016;7:e2282–e2282.

152. Yi JJ, Barnes AP, Hand R, Polleux F, Ehlers MD. TGF-β Signaling Specifies Axons during Brain Development. Cell. 2010;142:144–157.

153. Hiew L-F, Poon C-H, You H-Z, Lim L-W. TGF-β/Smad Signalling in Neurogenesis: Implications for Neuropsychiatric Diseases. Cells. 2021;10:1382.

154. Kang T, Han Z, Zhu L, Cao B. TFR1 knockdown alleviates iron overload and mitochondrial dysfunction during neural differentiation of Alzheimer’s disease-derived induced pluripotent stem cells by interacting with GSK3B. Eur J Med Res. 2024;29:101.

155. Hur E-M, Zhou F-Q. GSK3 signaling in neural development. Nat Rev Neurosci. 2010;11:539–551.

156. Insel BJ, Schaefer CA, McKeague IW, Susser ES, Brown AS. Maternal iron deficiency and the risk of schizophrenia in offspring. Arch Gen Psychiatry. 2008;65:1136–1144.

157. McCann JC, Ames BN. An overview of evidence for a causal relation between iron deficiency during development and deficits in cognitive or behavioral function. Am J Clin Nutr. 2007;85:931–945.

158. Sørensen HJ, Nielsen PR, Pedersen CB, Mortensen PB. Association between prepartum maternal iron deficiency and offspring risk of schizophrenia: population-based cohort study with linkage of Danish national registers. Schizophr Bull. 2011;37:982–987.

159. Jorgenson LA, Wobken JD, Georgieff MK. Perinatal iron deficiency alters apical dendritic growth in hippocampal CA1 pyramidal neurons. Dev Neurosci. 2003;25:412–420.

160. Iron Deficiency Impairs Developing Hippocampal Neuron Gene Expression, Energy Metabolism, and Dendrite Complexity | Developmental Neuroscience | Karger Publishers. https://karger-com.ezproxy.lib.vt.edu/dne/article/38/4/264/326980/Iron-Deficiency-Impairs-Developing-Hippocampal. Accessed 30 March 2026.

161. Carlson ES, Tkac I, Magid R, O’Connor MB, Andrews NC, Schallert T, et al. Iron is essential for neuron development and memory function in mouse hippocampus. J Nutr. 2009;139:672–679.

162. Seligman PA, Butler CD, Massey EJ, Kaur JA, Brown JP, Plowman GD, et al. The p97 antigen is mapped to the q24-qter region of chromosome 3; the same region as the transferrin receptor. Am J Hum Genet. 1986;38:540–548.

163. Singh CSB, Eyford BA, Abraham T, Munro L, Choi KB, Okon M, et al. Discovery of a Highly Conserved Peptide in the Iron Transporter Melanotransferrin that Traverses an Intact Blood Brain Barrier and Localizes in Neural Cells. Front Neurosci. 2021;15.

164. Suryo Rahmanto Y, Bal S, Loh KH, Yu Y, Richardson DR. Melanotransferrin: search for a function. Biochim Biophys Acta. 2012;1820:237–243.

165. van Deventer S, Arp AB, van Spriel AB. Dynamic Plasma Membrane Organization: A Complex Symphony. Trends Cell Biol. 2021;31:119–129.

166. Park S, Heo JS, Mizuno S, Kim M, An H, Hong E, et al. Tm4sf19 deficiency inhibits osteoclast multinucleation and prevents bone loss. Metabolism. 2024;151:155746.

167. Choi C, Jeong YL, Park K-M, Kim M, Kim S, Jo H, et al. TM4SF19-mediated control of lysosomal activity in macrophages contributes to obesity-induced inflammation and metabolic dysfunction. Nat Commun. 2024;15:2779.

168. Li J, Wang L, Guo H, Shi L, Zhang K, Tang M, et al. Targeted sequencing and functional analysis reveal brain-size-related genes and their networks in autism spectrum disorders. Mol Psychiatry. 2017;22:1282–1290.

169. Noubade R, Wong K, Ota N, Rutz S, Eidenschenk C, Valdez PA, et al. NRROS negatively regulates reactive oxygen species during host defence and autoimmunity. Nature. 2014;509:235–239.

170. Wong K, Noubade R, Manzanillo P, Ota N, Foreman O, Hackney JA, et al. Mice deficient in NRROS show abnormal microglial development and neurological disorders. Nat Immunol. 2017;18:633–641.

171. Smith C, McColl BW, Patir A, Barrington J, Armishaw J, Clarke A, et al. Biallelic mutations in NRROS cause an early onset lethal microgliopathy. Acta Neuropathol (Berl). 2020;139:947–951.

172. Dong X, Tan NB, Howell KB, Barresi S, Freeman JL, Vecchio D, et al. Bi-allelic LoF NRROS Variants Impairing Active TGF-β1 Delivery Cause a Severe Infantile-Onset Neurodegenerative Condition with Intracranial Calcification. Am J Hum Genet. 2020;106:559–569.

173. Tsurumi Y, Hamada Y, Katoh Y, Nakayama K. Interactions of the dynein-2 intermediate chain WDR34 with the light chains are required for ciliary retrograde protein trafficking. Mol Biol Cell. 2019;30:658–670.

174. Topcu V, Yildirim SF, Turan HM. Reanalysis of Whole-Exome Sequencing Data of an Infant with Suspected Diagnosis of Jeune Syndrome Revealed a Likely Pathogenic Variant in GRK2: A Newly Associated Gene for Jeune Syndrome Phenotype. Mol Syndromol. 2024;15:119–124.

175. Baujat G, Huber C, El Hokayem J, Caumes R, Do Ngoc Thanh C, David A, et al. Asphyxiating thoracic dysplasia: clinical and molecular review of 39 families. J Med Genet. 2013;50:91–98.

176. Schmidts M, Hou Y, Cortés CR, Mans DA, Huber C, Boldt K, et al. TCTEX1D2 mutations underlie Jeune asphyxiating thoracic dystrophy with impaired retrograde intraflagellar transport. Nat Commun. 2015;6:7074.

177. Nishijima Y, Hagiya Y, Kubo T, Takei R, Katoh Y, Nakayama K. RABL2 interacts with the intraflagellar transport-B complex and CEP19 and participates in ciliary assembly. Mol Biol Cell. 2017;28:1652–1666.

178. Shalata A, Ramirez MC, Desnick RJ, Priedigkeit N, Buettner C, Lindtner C, et al. Morbid Obesity Resulting from Inactivation of the Ciliary Protein CEP19 in Humans and Mice. Am J Hum Genet. 2013;93:1061–1071.

179. Yıldız Bölükbaşı E, Mumtaz S, Afzal M, Woehlbier U, Malik S, Tolun A. Homozygous mutation in CEP19, a gene mutated in morbid obesity, in Bardet-Biedl syndrome with predominant postaxial polydactyly. J Med Genet. 2018;55:189.

180. Izaurralde E, Lewis J, McGuigan C, Jankowska M, Darzynkiewicz E, Mattaj IW. A nuclear cap binding protein complex involved in pre-mRNA splicing. Cell. 1994;78:657–668.

181. Rambout X, Maquat LE. The nuclear cap-binding complex as choreographer of gene transcription and pre-mRNA processing. Genes Dev. 2020;34:1113–1127.

182. Kataoka N. The Nuclear Cap-Binding Complex, a multitasking binding partner of RNA polymerase II transcripts. J Biochem (Tokyo). 2023;175:9–15.

183. Gebhardt A, Habjan M, Benda C, Meiler A, Haas DA, Hein MY, et al. mRNA export through an additional cap-binding complex consisting of NCBP1 and NCBP3. Nat Commun. 2015;6:8192.

184. Kataoka N, Ohno M, Moda I, Shimura Y. Identification of the factors that interact with NCBP, an 80 kDa nuclear cap binding protein. Nucleic Acids Res. 1995;23:3638–3641.

185. Dou Y, Barbosa I, Jiang H, Iasillo C, Molloy KR, Schulze WM, et al. NCBP3 positively impacts mRNA biogenesis. Nucleic Acids Res. 2020;48:10413–10427.

186. Groza T, Gomez FL, Mashhadi HH, Muñoz-Fuentes V, Gunes O, Wilson R, et al. The International Mouse Phenotyping Consortium: comprehensive knockout phenotyping underpinning the study of human disease. Nucleic Acids Res. 2023;51:D1038–D1045.

187. Kugeratski FG, Atkinson SJ, Neilson LJ, Lilla S, Knight JRP, Serneels J, et al. Hypoxic cancer-associated fibroblasts increase NCBP2-AS2/HIAR to promote endothelial sprouting through enhanced VEGF signaling. Sci Signal. 2019;12:eaan8247.

188. Medina A, Mahjoub Y, Shaver L, Pringsheim T. Prevalence and Incidence of Huntington’s Disease: An Updated Systematic Review and Meta-Analysis. Mov Disord Off J Mov Disord Soc. 2022;37:2327–2335.

189. Petriti U, Dudman DC, Scosyrev E, Lopez-Leon S. Global prevalence of Rett syndrome: systematic review and meta-analysis. Syst Rev. 2023;12:5.

190. Massier M, Doco-Fenzy M, Egloff M, Le Guillou X, Le Guyader G, Redon S, et al. 3q29 duplications: A cohort of 46 patients and a literature review. Am J Med Genet A. 2024;194:e63531.

191. Lange C, Turrero Garcia M, Decimo I, Bifari F, Eelen G, Quaegebeur A, et al. Relief of hypoxia by angiogenesis promotes neural stem cell differentiation by targeting glycolysis. EMBO J. 2016;35:924–941.

192. Sefik E, Duan K, Li Y, Sholar B, Evans L, Pincus J, et al. Structural deviations of the posterior fossa and the cerebellum and their cognitive links in a neurodevelopmental deletion syndrome. Mol Psychiatry. 2024. 14 May 2024. https://doi.org/10.1038/s41380-024-02584-8.

193. Jeyifous O, Waites CL, Specht CG, Fujisawa S, Schubert M, Lin E, et al. SAP97 and CASK mediate sorting of N-Methyl-D-Aspartate Receptors through a novel secretory pathway. Nat Neurosci. 2009;12:1011–1019.

194. Najm J, Horn D, Wimplinger I, Golden JA, Chizhikov VV, Sudi J, et al. Mutations of CASK cause an X-linked brain malformation phenotype with microcephaly and hypoplasia of the brainstem and cerebellum. Nat Genet. 2008;40:1065–1067.

195. Haldipur P, Millen KJ, Aldinger KA. Human Cerebellar Development and Transcriptomics: Implications for Neurodevelopmental Disorders. Annu Rev Neurosci. 2022;45:515–531.

196. CRISPR-based functional genomics for neurological disease | Nature Reviews Neurology. https://www.nature.com/articles/s41582-020-0373-z. Accessed 19 March 2026.

197. Harima R, Hara K, Tanemura K. TCTEX1D2 is essential for sperm flagellum formation in mice. Sci Rep. 2025;15:2413.

198. Bohgaki T, Bohgaki M, Cardoso R, Panier S, Zeegers D, Li L, et al. Genomic instability, defective spermatogenesis, immunodeficiency, and cancer in a mouse model of the RIDDLE syndrome. PLoS Genet. 2011;7:e1001381.

199. Humphries LA, Shaffer MH, Sacirbegovic F, Tomassian T, McMahon K-A, Humbert PO, et al. Characterization of in vivo Dlg1 deletion on T cell development and function. PloS One. 2012;7:e45276.

**Figure Legends**

**Figure 1. Functional categorization of 3q29Del locus genes.** The genes in the 3q29Del locus are displayed in categories assigned based on known protein functions.

**Figure 2. Mutational constraint and brain expression of 3q29Del genes.** (**a**) The Loss-of-function Observed/Expected Upper bound Fraction (LOEUF) is plotted for each 3q29Del locus gene arranged by chromosomal position (centromere to telomere) from the Genome Aggregation Database (gnomAD) version 4.1. Asterisks indicate brain expression ascertained from BrainSpan [53] (**b**). Data in **b** are mean RPKM +/- SEM across brain region samples and individuals from BrainSpan.

**Table 1**

| **Gene** | **LOEUF (v4.1.0)** | **pHaplo^1^** | **Brain Expression** | **Homozygous Mouse Phenotype** | **Driver Likelihood** |
| --- | --- | --- | --- | --- | --- |
| **TFRC** | 0.489 | 0.888 | +++ | Lethal, E12.5 [150] | **Tier 1** |
| ZDHHC19 | 1.205 | 0.262 | - | Male infertility [132] | No evidence |
| SLC51A | 0.924 | 0.773 | - | Disrupted bile biosynthesis [135] | No evidence |
| PCYT1A | 0.934 | 0.838 | ++ | Lethal, E3.5 [144] | Tier 2 |
| DYNLT2B | 1.236 | NA | +++ | Male infertility [197] | Tier 2 |
| TM4SF19 | 1.102 | 0.566 | + | Alterations in adipocytes [167] | Tier 3 |
| **UBXN7** | 0.276 | 0.979 | +++ | Lethal, pre-weaning (IMPC; [186]) | **Tier 1** |
| RNF168 | 1.406 | 0.802 | ++ | Immunodeficiency, multi-system effects [198] | Tier 2 |
| SMCO1 | 1.523 | 0.577 | - | Unknown | No evidence |
| WDR53 | 1.109 | 0.588 | + | Altered metabolism (IMPC; [186]) | No evidence |
| **FBXO45** | 0.395 | 0.966 | +++ | Lethal, perinatal [62] | **Tier 1** |
| NRROS | 0.909 | 0.819 | + | Reduced lifespan [170] | Tier 3 |
| PIGX | 1.737 | 0.461 | +++ | Lethal, E12.5 (IMPC; [186]) | Tier 2 |
| CEP19 | 1.208 | 0.348 | ++ | Obesity, metabolic syndrome [178] | Tier 3 |
| **PAK2** | 0.662 | 0.963 | +++ | Lethal, E12.5 (IMPC; [186]) | **Tier 1** |
| SENP5 | 0.458 | 0.973 | +++ | No significant phenotypes (IMPC; [186]) | Tier 2 |
| **NCBP2** | 0.664 | 0.899 | +++ | Lethal, E9.5 (IMPC; [186]) | **Tier 1** |
| NCBP2AS2 | NA | NA | - | Unknown | No evidence |
| PIGZ | 1.431 | 0.747 | ++ | Altered metabolism (IMPC; [186]) | Tier 2 |
| MELTF | 1.039 | NA | + | Unknown | No evidence |
| **DLG1** | 0.491 | 0.993 | +++ | Lethal, perinatal [199] | **Tier 1** |
| BDH1 | 1.188 | 0.811 | ++ | Lethal, perinatal, incomplete penetrance [146] | Tier 2 |

^1^From Collins et al. 2022, red indicates a value greater than 0.86, which is a CNV-based haploinsufficiency threshold computed based on protein-truncating variant constrained genes.


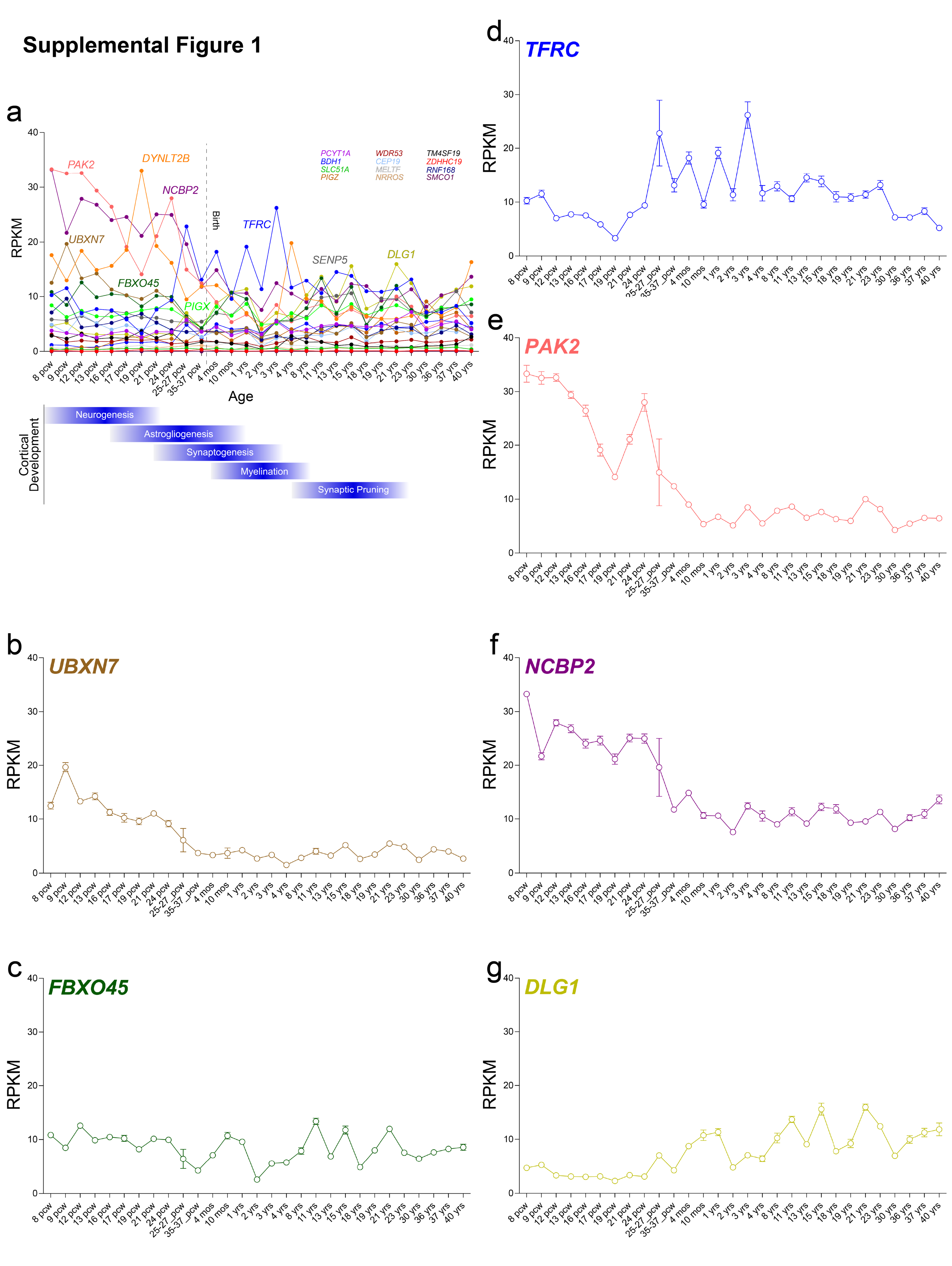


**Supplemental Figure 1. BrainSpan expression data for 3q29 genes.** (**a**) All 3q29 genes included in BrainSpan (top) with key phases of human cortical development illustrated below the x-axis timeline. (**b**-**g**) Individual expression traces for 3q29 Tier 1 genes.
